# Using quantile regression and relative entropy to assess the period of anomalous behavior of marine mammals following tagging

**DOI:** 10.1002/ece3.9967

**Published:** 2023-04-10

**Authors:** Lars Reiter Nielsen, Outi M. Tervo, Susanna B. Blackwell, Mads Peter Heide‐Jørgensen, Susanne Ditlevsen

**Affiliations:** ^1^ Data Science Laboratory Department of Mathematical Sciences University of Copenhagen Copenhagen Denmark; ^2^ Greenland Institute of Natural Resources Nuuk Greenland; ^3^ Greenland Institute of Natural Resources Copenhagen Denmark; ^4^ Greeneridge Sciences, Inc. SantaBarbara California USA

**Keywords:** accelerometer data, behavioral analysis, biologging of whales, ecological methods, quantile regression, relative entropy, tagging effects

## Abstract

Tagging of animals induces a variable stress response which following release will obscure *natural* behavior. It is of scientific relevance to establish methods that assess recovery from such behavioral perturbation and generalize well to a broad range of animals, while maintaining model transparency. We propose two methods that allow for subdivision of animals based on covariates, and illustrate their use on N=20 narwhals (*Monodon monoceros*) and N=4 bowhead whales (*Balaena mysticetus*), captured and instrumented with Acousonde™ behavioral tags, but with a framework that easily generalizes to other marine animals and sampling units. The narwhals were divided into two groups based on handling time, *short* (t<58 min) and *long* (t≥58 min), to measure the effect on recovery. Proxies for energy expenditure (VeDBA) and rapid movement (jerk) were derived from accelerometer data. Diving profiles were characterized using two metrics (target depth and dive duration) derived from depth data. For accelerometer data, recovery was estimated using quantile regression (QR) on the log‐transformed response, whereas depth data were addressed using relative entropy (RE) between hourly distributions of dive duration (partitioned into three target depth ranges) and the long‐term average distribution. Quantile regression was used to address location‐based behavior to accommodate distributional shifts anticipated in aquatic locomotion. For all narwhals, we found fast recovery in the tail of the distribution (<3 h) compared with a variable recovery at the median (∼1–10 h) and with a significant difference between groups separated by handling time. Estimates of bowhead whale recovery times showed fast median recovery (<3 h) and slow recovery at the tail (>6 h), but were affected by substantial uncertainty. For the diving profiles, as characterized by the component pair (target depth, dive duration), the recovery was slower (narwhals‐*long*: t<16 h; narwhals‐*short*: t<10 h; bowhead whales: <9 h) and with a difference between narwhals with short vs long handling times. Using simple statistical concepts, we have presented two transparent and general methods for analyzing high‐resolution time series data from marine animals, addressing energy expenditure, activity, and diving behavior, and which allows for comparison between groups of animals based on well‐defined covariates.

## Graphical abstract

We propose two methods to assess recovery from behavioural perturbations related to tagging of marine animals, and illustrate the techniques on narwhals and bowhead whales.

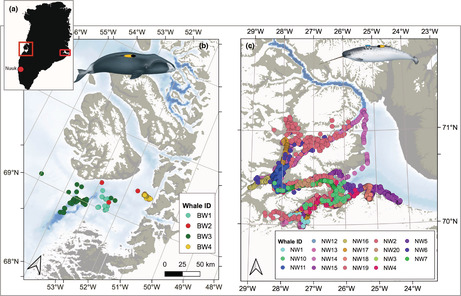

## 1 INTRODUCTION

In the last decade, published journal articles relating to cetacean tagging have accelerated (Andrews et al., 2019). With smaller and more advanced telemetry and biologging devices (Lennox et al., 2017), it is now possible to understand movement ecology in unprecedented ways, which has attracted a lot of attention from researchers around the world. One key benefit of animal‐borne technology is the ability to monitor certain marine mammals that would otherwise be problematic, due to either untraceable migration patterns or remote lifestyles in inaccessible domains (Heide‐Jørgensen et al., 2008; Lydersen et al., 2020). By attaching multisensor biologgers to marine animals, researchers can collect a wide range of data on the physiology, behavior, and ecology of marine species with high temporal resolution of a few seconds to minutes. The data collected through biologging include the location and movement of the animal, which is typically tracked by GPS or other forms of telemetry. Data on diving behavior can also be obtained through tri‐axial accelerometer measurements, allowing researchers to track the activity levels of the animal. Additionally, biologgers can be used to measure various physiological parameters, such as heart rate and body temperature, as well as environmental factors, such as temperature, salinity, and depth (Hussey et al., 2015; Wilmers et al., 2015).

Numerous studies have utilized such biologging devices to give fascinating insight into aspects of aquatic locomotion using various statistical and machine learning methods. For example, a popular approach to understand individual movement is to identify latent states corresponding to different activities using hidden Markov models (Adam et al., 2019; DeRuiter et al., 2017; Leos‐Barajas et al., 2017; Ngô et al., 2019) while other studies have used biologgers to investigate the effect of anthropogenic perturbations on marine wildlife (Heide‐Jørgensen et al., 2021; Miller et al., 2015; Sivle et al., 2016; Tervo, Blackwell, et al., 2021). These are just a few examples of how ecologists can benefit from biologging.

In contrast to the benefits, concerns have also been raised about the effect of tagging on animals (Andrews et al., 2019; Batsleer et al., 2020; Lennox et al., 2017; Todd Jones et al., 2013). Capture and tagging will to some extent jeopardize the health and well‐being of said animal, and induce a variable stress response (Andrews et al., 2019; Williams, Blackwell, et al., 2017). It goes without saying that stress should be minimized due to welfare issues, but from a pragmatic point of view, this is also desirable, since the goal normally is to collect data that are not affected by aberrant behavior from stress associated with tagging. A conservative choice is to trim the sampled data at a point in time, where one is *convinced* that the animal has resumed to unaffected behavior. However, this requires a priori knowledge about the effect of tagging relative to the animal in question, in order to not underestimate the duration of anomalous behavior. An animal may appear to have recovered in certain metrics such as fine‐scale movement, but still be far from baseline behavior in other metrics, such as depth‐related ones. On the contrary, overestimating the time of recovery is equally problematic, as a significant portion of informative data will be lost. To ensure that these data are used efficiently and the potential impact of tagging on the datasets is minimized, it is therefore crucial to determine the time of recovery with high degree of confidence.

It is also noteworthy that the utilization of the tag can potentially compromise the validity of the data, even when the stress response elicited by the act of tagging has dissipated (Batsleer et al., 2020; Todd Jones et al., 2013; Walker et al., 2012) For example, it is necessary to account for the drag effect that may be imposed by tags with high mass‐ratio relative to the animal in question, since this may alter the behavior of the animal (Todd Jones et al., 2013). Because of this, it has been deemed important to identify and adopt a “best practice” with respect to capture and tagging methods (Walker et al., 2012), such that immediate and long‐term effects are minimized. In this paper, we assume that there is only a transient effect of any capture, tagging, and release protocol. The objective of this study was to propose general methods that establish the time of recovery from tagging of marine animals, thereby eliminating the need for more or less arbitrary data trimming.

Previous studies have leaned on mean‐based regression (MR) models as a tool to assess recovery of diving behavior (Shuert et al., 2021; van Beest et al., 2018). In Shuert et al. (2021), the long‐term mean was subtracted from hourly mean values over various movement measures, and a best‐fit model was selected from a set of generalized additive models based on the AIC‐criterion. The time of recovery was then defined as the point in time when mean estimates were no longer significantly different from zero. While this procedure captures the variability of the data nicely, it is also prone to overfitting. Furthermore, the lack of monotonicity of the evolution of the mean estimates makes the time of recovery ambiguous, as the null‐hypothesis (mean is zero) will potentially be accepted at some time point, and rejected the next. Similar shortcomings appeared in van Beest et al.'s (2018) study, where the authors estimated the component (of several movement metrics) ascribed to individual variability using generalized additive models. This component was then subtracted from the original measurements to obtain movement baseline values. Finally, segmented regression was applied to the baseline values, and the number of breakpoints was determined based on AIC score. The initial breakpoint describes the transition between the perturbed behavioral state (due to tagging) and natural behavior, while the causal explanation of the remaining change points was not identified in this study, but could be other type of disturbances (see Heide‐Jørgensen et al. (2021)). A potential problem with this approach is that target metrics likely vary in distribution over different activities. Thus, the interpretation of a change point as a shift back to natural behavior might not be verifiable. This activity dependency makes it problematic to declare a single baseline behavior. Instead, the authors estimate the time it takes for the animal to return to normal behavior (hereafter called “return time”), averaged over all activities. In fact, this is an inherent issue with MR when it comes to animals, as their behavior at a fine scale is likely to change across different activities (Cade & Noon, 2003).

To address these problems, we propose a quantile‐based regression (QR) approach as a more robust alternative for accelerometer data, specifically vectorial dynamic body acceleration (VeDBA) and jerk describing fine‐scale movement (both metrics are defined in Section 2.2, Equations 1 and 2). In the QR approach, covariates are chosen a priori based on domain knowledge, and fitted values are restricted to a monotonic pattern. While MR measures the overall recovery, QR allows for the differentiation between various locations within the distribution. For example, QR using the median describes typical activities, whereas QR using, say, the 90% quantile, describes atypical activities. In both above studies (Shuert et al., 2021; van Beest et al., 2018), depth data were analyzed in similar ways as accelerometer data (in terms of VeDBA and jerk) using MR on depth‐derived metrics, such as mean depth, maximum depth (*target depth*), and dive duration. Here, we suggest characterizing a dive by simultaneously including target depth and dive duration because we believe they give a more accurate representation of a dive, and subsequently determining when the pair is normalized by applying the concept of relative entropy (RE) and a heuristic approach. As with QR, this approach also allows for subdivision of animals based on covariates. To ensure a nonambiguous return time, we construct a long‐term confidence band (for the entropies) denoted *region of recovery* (RoR). A voting system is then introduced by transforming the entropies to labels with values +1 and −1 depending on whether they are inside or outside the RoR, respectively. By performing segmented regression on the cumulated sum of labels, we then estimate the time at which the diving profile is stabilized within the RoR.

We illustrate the methods on high‐resolution accelerometer and depth data of N=20 East Greenland narwhals (*Monodon monoceros*) and N=4 West Greenland bowhead whales (*Balaena mysticetus*) and estimate the return time to normal behavior following release, which is then compared with the results found using a MR framework as described by Shuert et al. (2021). The techniques are, however, quite general and easily applicable to other aquatic animals. The presented methods are flexible, in the sense that they allow for the study animals to be split into groups with specific covariates that bear scientific relevance. For example, a study exploring the behavioral response from tagging on migrating humpback whales (*Megaptera novaeangliae*) found that disturbance of female‐calf groups was greater than that of other age/sex groups (Williamson et al., 2016). A sensible split of the humpback whales could be based on such a marker. We illustrate this flexibility by dividing the narwhals into groups based on handling time, whereas the bowhead whales are not split (due to a low sample size).

## 2 MATERIALS AND METHODS

### 2.1 Capture and tagging

#### 2.1.1 Narwhals

**FIGURE 1:**
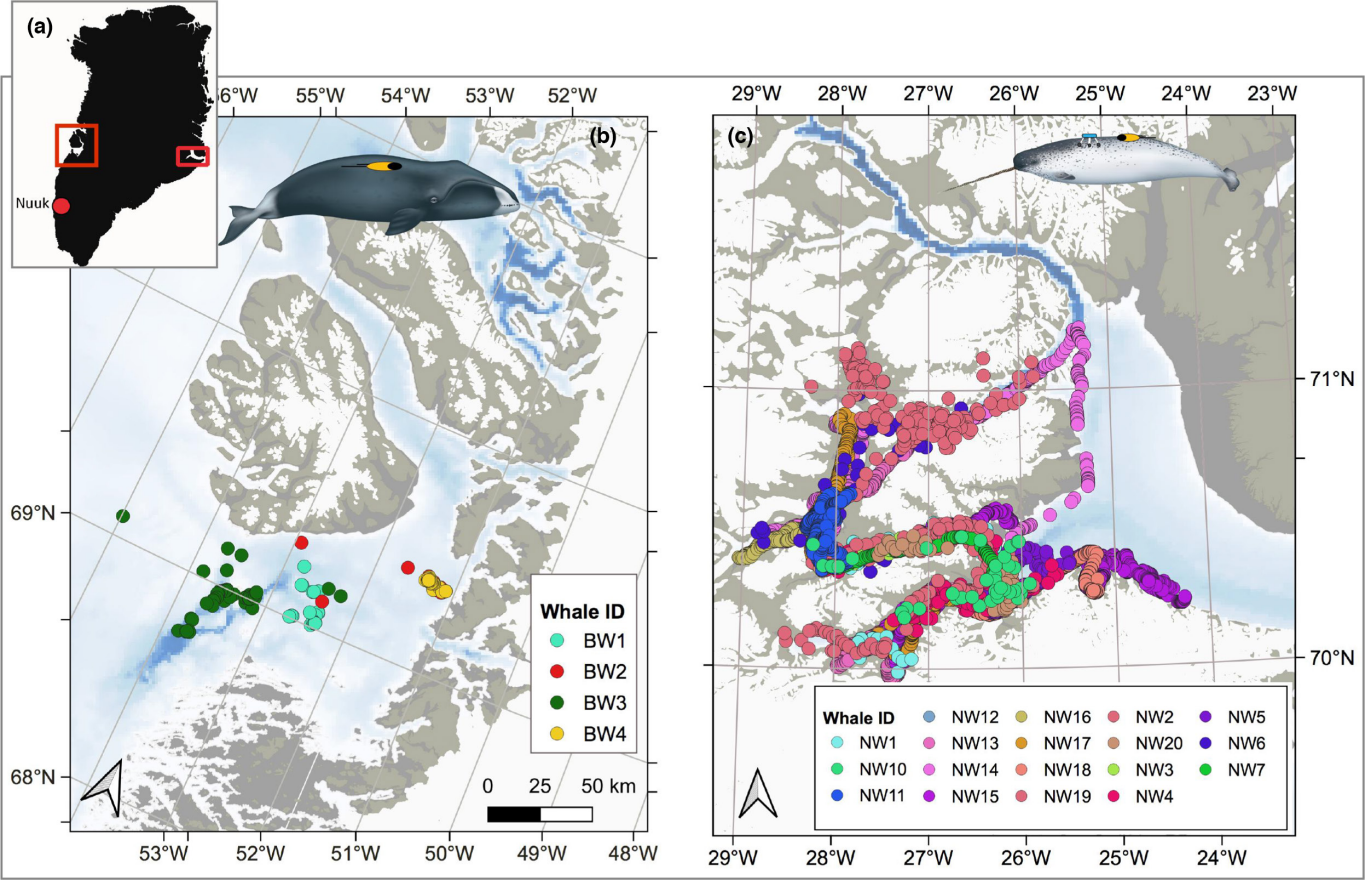
(a) Map of Greenland, showing Disko Bay (left red square) where bowhead whales were tagged and Scorseby Sound (right red square) where narwhals were captured and tagged. Closeup of Disko Bay (b) and Scoresby Sound (c) with GPS locations of whales shown with colored dots.

We analyzed data from N=20 narwhals (Table 1) captured in Scoresby Sound, East Greenland (Blackwell et al., 2018; Heide‐Jørgensen et al., 2015; Tervo, Ditlevsen, et al., 2021) and monitored during the summer seasons ranging from 2013 to 2018 as part of an ongoing study of narwhal ecology and behavior (Heide‐Jørgensen et al., 2020). The location data are illustrated in Figure 1.

**TABLE 1:** Characteristics of the N=20 East Greenland narwhals (NW) and N=4 West Greenland bowhead whales (BW) analyzed in this paper. NW3 and NW9 (marked with a *) are only included in the depth analysis, since the accelerometer data were sampled at a lower frequency. NW7 had an estimated handling time below 58 min.

Whale	Sex	Ht. (minutes)	Ht. id	Length (cm)	Sd. (hours)	Tags/units
NW1	M	18	Short	409	103	A/G/H
NW2	M	30	Short	470	103	A/G
NW3*	F	34	Short	380	83	A/G
NW4	M	36	Short	487	195	A/G/H
NW5	M	37	Short	510	201	A/G/S
NW6	M	41	Short	460	150	A/G
NW7	F	X	Short	390	11	A/G
NW8	F	58	Long	341	103	A/G
NW9*	F	60	Long	420	65	A/G
NW10	M	61	Long	457	103	A/G
NW11	M	62	Long	410	111	A/G
NW12	M	64	Long	372	192	A/G
NW13	M	69	Long	454	13	A/G/H
NW14	M	70	Long	330	35	A/G
NW15	F	71	Long	393	207	A/G
NW16	M	73	Long	436	194	A/G
NW17	F	80	Long	379	56	A/G
NW18	M	81	Long	497	202	A/G/H
NW19	F	88	Long	465	102	A/G
NW20	F	90	Long	360	103	A/G
BW1	M	‐	‐	‐	24	A
BW2	M	‐	‐	‐	20	A
BW3	F	‐	‐	‐	14	A
BW4	F	‐	‐	‐	8	A

*Note*: Ht., Handlingtime; Sd., Sampling duration; A, Acousonde; G, GPS backpack; H, (HTR) Heart Rate Monitor; S, Speedometer.

The whales were captured using set nets of length 40 or 80 metres stretching from shore to an anchor. The nets were kept under surveillance at all times. Whenever a whale was spotted, fiberglass speed boats were used to herd the whales into the nets, and once a whale was succesfully entangled the net was released from its anchor and pulled to shore (Heide‐Jørgensen et al., 2015; Tervo, Ditlevsen, et al., 2021). Instrumentation with tags took place near shore, while the whale was afloat in shallow water, flanked by four to six handlers in survival suits, with a priority of minimizing handling time.

All whales were instrumented with a backpack‐satellite transmitter from Wildlife Computers and an Acousonde™ sound and movement tag (Acoustimetrics, http://www.acousonde.com), for details, see Tervo, Ditlevsen, et al. (2021); Heide‐Jørgensen et al. (2015). The satellite tag was attached to the back of the whales using sterilized delrin nylon pins of length 8 mm. The Acousonde tag was attached to the side of the dorsal ridge using suction cups. A few whales (see Table 1) were instrumented with additional tagging units collecting data not used in this paper. The data used in this paper include depth data (sf 10 Hz) and tri‐axial accelerometer data (sf 100 Hz). Following detachment (See Table 1 for deployment duration), Acousondes were located using signals from an Argos Transmitter (Wildlife Computers SPOT5) and a VHS transmitter (ATS telemetry), attached to each Acousonde tag (Blackwell et al., 2018). Tag size and hydrodynamic shape were minimized such that the tags represented less than 3% of the frontal area ratio of the whale, thus reducing the effect of drag (Tervo, Ditlevsen, et al., 2021; Todd Jones et al., 2013). In this study, we therefore assumed that any effect relating to the tag is either negligible or transient.

#### 2.1.2 Bowhead whales

We analyzed data from N=4 bowhead whales tagged in Disko Bay, Greenland, in April 2013 as part of a project carried out by the Greenland Institute of Natural Resources. Tagging was performed from small vessels, owned and operated by local hunters of the village of Qeqertarsuaq, on Disko Island (Figure 1).

Whales were pursued for 30–45 min before tagging, which was done using an 8‐m fiberglass pole as described by Heide‐Jørgensen et al. (2003). The Acousonde tag was tethered to a 4‐cm stainless steel spear, which was implanted under the skin, 10 cm into the blubber, in the upper third of the whale's back. A magnesium link was connected to the tether right above the skin. This link corroded in the presence of salt water and detached the tag from the insertion point after a period of time. See Table 1 for deployment duration. A biopsy was taken by the tagging pole during the tagging event, allowing genetic sexing of the animals.

#### 2.1.3 Permission

This study is part of the Northeast Greenland Environmental Study Program, which is a collaboration between DCE—Danish Centre for Environment and Energy at Aarhus University, the Greenland Institute of Natural Resources, and the Environmental Agency for Mineral Resource Activities of the Government of Greenland. Permission for capturing, handling, and tagging of narwhals was provided by the Government of Greenland (Case ID 2010 ± 035453, document number 429926). The project was reviewed and approved by the IACUC of the University of Copenhagen (17 June 2015). Access and permits to use land facilities in Scoresby Sound were provided by the Government of Greenland. No protected species were sampled. The tagging of bowhead whales was conducted under the general permission from the Greenland Government to the Greenland Institute of Natural Resources.

### 2.2 Preprocessing steps

To allow direct comparison of our results from the narwhal study with those obtained using the method proposed for narwhals by Shuert et al. (2021), we preprocessed the data in the same way; thus, the depth and accelerometer data streams collected by the Acousondes on narwhals were either up‐ or downsampled to match the 50 Hz sampling rate used by Shuert et al. (2021). Consecutive measurements were averaged for downsampling while measurements were duplicated, as necessary, for upsampling. For the bowhead whales, both accelerometer and depth data were sampled at a frequency of 10 Hz for three of the whales and 5 Hz for the fourth. For the first three bowhead whales with higher sampling frequencies, we downsampled the data to 5 Hz by averaging over two consecutive measurements.

Prior to down‐ and upsampling the (raw) dive data of the whales (both species), we applied a *Luques filter* (Luque & Fried, 2011) to zero‐offset correct depth measurements, thus reducing inconsistency between recorded depth and actual depth (this drift is due to the temperature sensitivity of the pressure transducer).

The filtering method involved recursive smoothing based on moving quantiles. While the recursion in theory continues indefinitely, in practice a two‐step filtering is often adequate (see also Luque and Fried (2011)). Higher quantiles are preferred when the signal is noisy, and as residual noise of the recursive output signals tend to decrease, so will our choice of quantiles. The objective of the first filter is the removal of noise from surface measurements. We used a median filter over a 20‐second moving window. The relatively narrow size of the window was chosen to avoid erosion of the surface signal. The second filter was then applied to the first filter to detect the correct surface level. Since the noise was minimal, we used the 0.01 quantile and a window size of 30 min to accommodate diving cycles of <30 min for both narwhals and bowhead whales. The adjusted depth was calculated by subtracting the output of the second filter from the first.

From the (adjusted and upsampled) depth data, we derived two metrics for diving behavior for both species, namely target depth and dive duration. Following the convention of previous studies (Ngô et al., 2019; Tervo, Ditlevsen, et al., 2021), we defined the surface area to be depths no greater than 20 meters. Target depth was then defined as the deepest depth attained between leaving and re‐entering the surface area. Dive duration was the time spent in any such dive.

As a proxy for energy expenditure, we used VeDBA (Shuert et al., 2021; Wilson et al., 2020):

(1) $$\text{VeDBA}={\left({\mathrm{DA}}_{x}^{2}+{\mathrm{DA}}_{y}^{2}+{\mathrm{DA}}_{z}^{2}\right)}^{1/2}$$

where $\mathrm{DA}\equiv \left({\mathrm{DA}}_{x},{\mathrm{DA}}_{y},{\mathrm{DA}}_{z}\right)$ is the dynamic acceleration. Dynamic acceleration was derived by taking the raw acceleration and subtracting the static component ascribed to gravity. To calculate static acceleration, we low‐pass filtered the acceleration data in each axis with a cutoff frequency of 0.1 Hz (Stothart et al., 2016; Williams, Holton, et al., 2017).

To estimate activity or rapid movement (Allen et al., 2016; Ydesen et al., 2014), we used the norm of the differential of raw acceleration $A\equiv \left(A_{x},A_{y},A_{z}\right)$ called *jerk*:

(2) $$\text{jerk}={\left(\partial A_{x}^{2}+\partial A_{y}^{2}+\partial A_{z}^{2}\right)}^{1/2}$$

**FIGURE 2:**
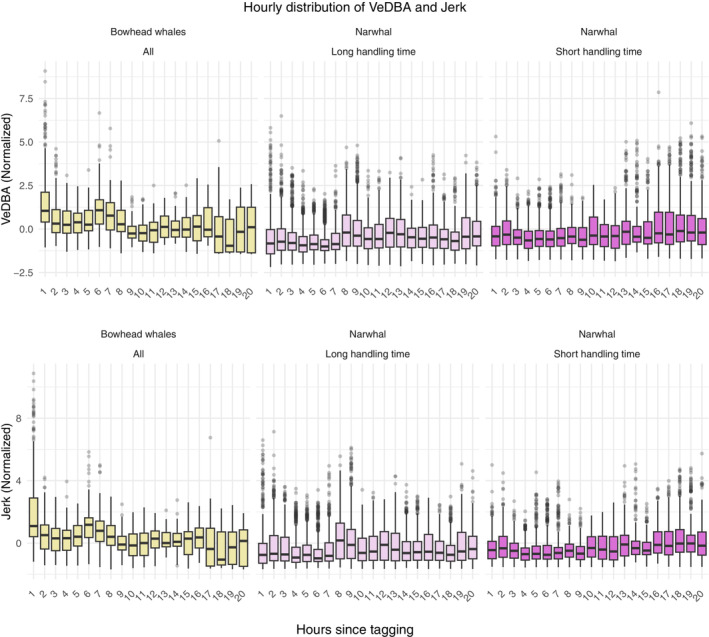
Energy and activity profiles for N=4 bowhead whales (left column) and N=18 narwhals (middle and right column) deduced from accelerometer data. Progression of hourly normalized VeDBA (top) and jerk (bottom) over the first 20 h of combined records for each category. Normalization is done by subtracting the population mean and dividing by the population standard deviation, both of these obtained in the late stage of each tracking period (>10 h for bowhead whales and >40 h for narwhals). At each hour, a boxplot showing 25th (Q1) and 75th (Q3) empirical quantiles (lower and upper edge of the box) as well as the median (solid line inside the box) are depicted along with outliers (points) defined as observations with a distance $>1.5\times \left(Q3-Q1\right)$ from the edges of the box. The narwhal sample are separated in long handling times (N=11; middle) and short handling times (N=7; right).

As a preliminary step, and to get a first indication of the presence of a transient stress response, hourly distributions of both metrics were visualized (Figure 2). After an hour since tagging, the activity profiles of the bowhead whales experienced a shift in distribution, where larger values became less common, indicating recovery in the tail of the distribution. The median, 25th quantile, and 75th quantile also seemed to quickly stabilize. For the narwhals, a slight shift in median was seen for both VeDBA and jerk after approximately 7 h in the group with long handling times, whereas the central behavior seemed unaffected for the group with short handling times, thus revealing a potential effect of handling time around the median. The width of the boxplots as well as the number of extreme observations (outliers) were also temporally constant, suggesting that behavior quickly recovered in the tail of the distribution.

### 2.3 Quantile regression on accelerometer data

To assess the recovery of energy‐ and activity‐based metrics (VeDBA and jerk), we fitted a QR model. Owing to the erratic behavior of wild animals, let alone the distributional shifts caused by changing activities, we obtained measurements with a huge range and irregular patterns (see for example Figure 2 and Appendix S1).

QR is useful in this respect, as model estimates are less influenced by extreme observations. In addition, QR allows one to focus on different locations of the distribution. Considering the response at the quantiles (and not mean response, as in ordinary least squares) improves the predictive capability as it accommodates for nonidentical slopes and uneven variance over different locations of the distribution possibly resulting from activity and environmental changes. QR, as opposed to ordinary least squares, is also more robust to an imperfect set of covariates. This is often the case in ecology, where variables of interests are expected to have complicated relationships to a large set of predictor variables (Cade & Noon, 2003). In relation to cetacean locomotion, a plausible set of (hidden) covariates presumably stem from individual activities and environmental effects (see for instance Gleiss et al. (2017)).

Let Y be the response (= jerk,VeDBA) and $Y^{\tau }$ be the τ‐quantile of Y, then we used the following QR model

(3) $$\log\left(Y^{\tau }\right)\;|\;X,t=\beta_{\mathrm{Ind}}+\alpha \left(\frac{1}{t+1}\right)$$

where $X\equiv \left\{\mathrm{Ind}\right\}\cup X_{0}$ are covariates, and always include a general individual effect Ind entering the model through the offset $\beta_{\mathrm{Ind}}$. The individual term could depend on covariates that are related to normal behavior, however, we did not pursue that in this paper. The time t indicates time since tagging measured in hours. Other covariates related to how fast the animal recovers from tagging could include *handling time*, *sex*, and *size* contained in $X_{0}$ that enters the model through the slope:

(4) $$\alpha =\alpha_{0}\theta^{T}X_{0}.$$

Here, θ is a vector of coefficients and ${}^{T}$ denotes the transpose. If there are no covariates, then $\alpha =\alpha_{0}$ is constant. In this study, we used $X_{0}=\left\{\text{Handlingtime}\right\}$ for the narwhals and no covariates for the bowhead whales.

Equation (3) implies

(5) $$Y^{\tau }=\beta_{\mathrm{Ind}}^{*}e^{\mathrm{\alpha z}}$$

where $z=\frac{1}{t+1}$ and $\beta_{\mathrm{Ind}}^{*}=e^{\beta_{\mathrm{Ind}}}$. Equation (5) consists of two factors. The first is an individual‐specific offset during normal behavior, and the second is an exponential decay term with a rate depending on the relevant covariates. At the release time t=0, then z=1 and the effect of all covariates is maximized. When t tends to infinity, $e^{\mathrm{\alpha z}}$ tends to one and the effect vanishes.

The pertinent question is then at what time t the response (at that quantile) changes less than some $\delta \in \left(0,1\right)$. This amounts to solving the following equation in z (see also Figure 4):

(6) $$e^{\mathrm{\alpha z}}=\delta .$$

We considered two quantiles, τ=0.5 and τ=0.9, to assess both central and tail behavior. QR was implemented in R using the Quantreg package (Koenker, 2022).

### 2.4 Relative entropy on depth data

Diving behavior can be characterized by target depth and dive duration. While there is an inherent correlation between these metrics (Laidre et al., 2002), the pair is not strictly collinear. For example, a whale could descend to some (target) depth and quickly ascend, or it could roam at the bottom before ascending. The first is known as a V‐dive and the latter as a U‐dive, and while they share the same target depth, they likely have different dive durations and biological functions.

To accommodate both components, distributions of dive duration discretized into categories of target depth were considered. For the narwhals, the categories were *shallow dives* (20–160 m), *medium dives* (160–360 m), and *deep dives* (>360 m). This partition was inspired by another study (Ngô et al., 2019), which recognized three typical dive depth categories. The medium and deep dives are associated with foraging. For the bowhead whales, the partition was based on known diving behavior from previous studies (Heide‐Jørgensen et al., 2013). The categories were *shallow dives* (20–60 m), *medium dives* (60–120 m), and *deep dives* (>120 m). For both species, the partition was also validated by calculating the (33rd and 66th) depth percentiles, and by eye‐balling frequency diagrams of the target depth. While other finer discretizations could work as well, we aimed to minimize the number of categories by only using the broadest depth ranges that are believed to be distinct in terms of diving activity.

Throughout the RE analysis, we used the concept of a *normal region*, in which whales are expected to have recovered. This was our initial guess of the time of recovery and is defined by a threshold value of time $t_{N}$, such that observations in the normal region $t>t_{N}$ reflected baseline behavior. We chose $t_{N}=40$ h for the narwhals and $t_{N}=10$ h for the bowhead whales. The selection was based on inspection of the target depth and dive duration pair of the study animals (a trimmed version is visualized in Figure 5), as well as domain knowledge. For the narwhals, the choice was also influenced by previous findings (Blackwell et al., 2018; Shuert et al., 2021).

Our approach estimates a time of recovery, which is likely (but not necessarily) smaller than $t_{N}$. We will return to this subtlety in the discussion. Even so, it is important that an appropriate value for $t_{N}$ is selected, balancing the requirement for a sufficient time to ensure recovery while also allowing for the utilization of data within the normal region ($t>t_{N}$) to determine a proper reference distribution Q, which is defined below.

In this method, hourly distributions of dive duration (relative to target depth) written $P_{t}\equiv \left(P_{t}^{\text{Shallow}},P_{t}^{\text{Medium}},P_{t}^{\text{Deep}}\right)$ were compared to a long‐term ($t\geq t_{N}$) weighted average distribution:

(7) $$Q\equiv \left(Q^{\text{Shallow}},Q^{\text{Medium}},Q^{\text{Deep}}\right)$$

(8) $$=\sum_{i=1}^{M}w_{i}\sum_{t=t_{i-1}}^{t_{i}}\frac{1}{t_{i}-t_{i-1}}P_{t}$$

where M counts the number of tags that were lost or malfunction in the normal region ranging from $t_{0}\equiv t_{N}$ to $t_{M}$ (= the maximum sample duration). These losses happened at times $t_{i},i=1,\ldots ,M$. The weights $w_{i}$ were chosen such that priority was given to the parts where most sampling devices were active. We chose the simple form $w_{i}=\frac{n_{i}}{\sum_{j=1}^{M}n_{j}}$, where $n_{i}$ was the number of active whales *before* drop point $t_{i}$. The motivation for introducing a weighting protocol was to exploit all observations in the normal region for statistical power when computing the reference distribution Q, while at the same time avoiding any potential bias resulting from a temporal decline in the sample of whales (see sampling durations in Table 1). The comparison between Q and $P_{t}$ was made using the Jensen–Shannon relative entropy (abbreviated RE) $J\left(\cdot ,\cdot \right):{\mathbb{R}}^{3}\times {\mathbb{R}}^{3}\mapsto \left[0,1\right]$ as our similarity measure, defined as

(9) $$J\left(P_{t},Q\right)=\frac{1}{2}K\left(P_{t},M\right)+\frac{1}{2}K\left(Q,M\right)$$

with $M=\frac{1}{2}\left(P_{t}+Q\right)$, $K\left(\cdot ,\cdot \right):{\mathbb{R}}^{3}\times {\mathbb{R}}^{3}\mapsto [0,\infty [$ being the Kullback–Leibler divergence defined as $K\left(P,Q\right)=\sum_{x\in X}P_{t}^{\left(x\right)}\log\left(\frac{P_{t}^{\left(x\right)}}{Q^{\left(x\right)}}\right)$ and $X=\left\{\text{Shallow},\text{Medium},\text{Deep}\right\}$.

$J\left(P_{t},Q\right)$ can be viewed as a measure of variation (or *divergence*) between $P_{t}$ and the expected distribution under normal behavior Q. We established when the RE was no longer significantly different from the expected RE within the normal region.

#### 2.4.1 Region of recovery

To establish when the RE was typical, a 95% confidence interval called the *region of recovery* (RoR) was constructed using a leave‐one‐out cross‐validation approach. We defined for all $t^{\prime }\geq t_{N}$ the objects $Q_{-t^{\prime }}=\sum_{i=1}^{M}w_{i}\sum_{\begin{array}{c} t=t_{i-1}\\ t\neq t^{\prime } \end{array}}^{t_{i}}\frac{1}{t_{i}-t_{i-1}}P_{t}$ and $J_{-t}=J\left(P_{t},Q_{-t}\right)$. Then from $\left(J_{-\left(t_{N}+1\right)},,J_{-T}\right)$ we extracted a 95% confidence interval for RE in the normal region ($t\geq t_{N}$). T was chosen among the first few drop points, to avoid altering the distribution of $P_{t}$ due to changing subset(s) of whales. We chose T=80 h for narwhals and T=20 h for bowhead whales.

Since $J\left(P_{t},Q\right)$ can leave and enter the RoR in an inconsistent manner and with a wide temporal range, a voting system was introduced. Whenever $J\left(P_{t},Q\right)$ was outside the RoR, it was assigned a value of −1, while a value inside the region got a +1. We then took the cumulative sum over these binary labels at each t and looked for a breakpoint using *block bootstrapping* (blocks with a temporal range of 5 h for narwhals, and 2 h for bowhead whales) and performed segmented regression (Muggeo, 2022) on the resulting data vector. This was taken as the return time to normal behavior (see Appendix S1 for details and for an example of bootstrap samples). The block size was chosen based on the rule $T^{1/3}$_,_ which has been proven to be a suitable choice (Hall et al., 1995). In the discussion section, we will further discuss the influence of T and the block size on our results.

#### 2.4.2 Comparing subgroups

To compare the effect between groups of animals (based on selected covariates), the method above was repeated for each group, and the hourly distributions $P_{t}$ were restricted to the individuals of the relevant group. The RoR was then constructed from the combined set of RE measurements from each group, where one assumes that the groups share a common distribution whenever $t\geq t_{N}$ (see also Sections 4 and 3.3). However, there is a caveat in the presented method. Whenever the monitored individuals are few in numbers, either within a group or in total, there is the possibility of huge intergroup variability ascribed to individual differences. A simple way of gauging the presence of such an individual effect is to compare several RoRs in two settings, namely by computing hourly distributions (1) *without* conditioning on handling time and (2) conditioned on handling time. If the confidence intervals are approximately of the same size, then we expect such an effect to be minimal. In the opposite case, the conclusions are potentially confounded by an individual effect. In particular, the covariates might not be the only drivers for any detected differences in return time.

## 3 RESULTS

We illustrate the methods on the N=20 East‐Greenland narwhals and N=4 West‐Greenland bowhead whales summarized in Table 1. The narwhals were split into two groups, with a short handling time (t<58 min; N=7, mean ∼33 min, range $\left[18,41\right]$ min) or long handling time (t≥58; N=13, mean ∼66 min, range $\left[58,90\right]$ min). In Section 3.1, the results are based on N=18 narwhals, since two narwhals had recorded accelerometer data sampled at a lower frequency compared with the other whales ( Table 1). We use minimization of within‐group variation and maximization of between‐group variation as our guiding principle in the division of narwhals into groups of handling time. The exact variation for the group of short handling time is, however, unknown due to the missing value of NW7.

### 3.1 Recovery of fine‐scale behavioral modes for N=18 narwhals and N=4 bowhead whales

**FIGURE 3:**
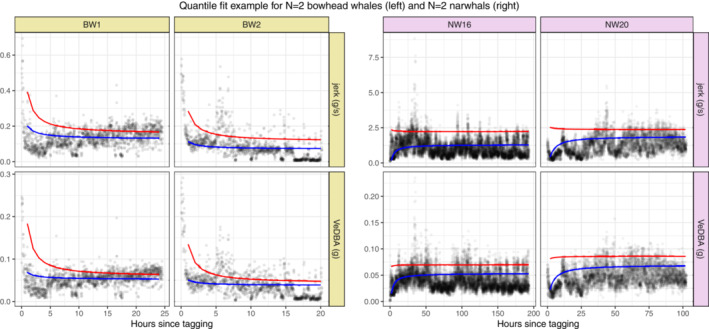
Quantile fit examples for bowhead whales (left) and narwhals (right), for jerk (top) and VeDBA (bottom). Observations (gray dots) are plotted for each minute along with Quantreg prediction (solid lines) for quantiles τ=0.5 (blue) and τ=0.9 (red). NW16 and NW20 both had long handling times.

In Figure 3, observations for selected whales are visualized along with the model fits (solid lines). Deviating fine‐scale behavior can be seen for all whales, as shown by the downward or upward shift in activity levels most pronounced immediately after release. While the overall trend seems to be traced nicely by the model, the data show a highly irregular pattern, especially in the beginning where the wavering motion has a complex composition, possibly resulting from an individual component and a component relating to activities. A tighter fit in the region of initial response might be obtained by adding an individual component to the (inverted) time covariate, but at the cost of less generality and increased uncertainty when measuring return times. For the selected bowhead whales (BW1 and BW2) in Figure 3, there seems to be a rapid decline in the tail of the distribution, whereas the change is much slower around the median. For the selected narwhals (NW16 and NW20), the return to normal behavior was almost instantaneous and much faster in the tail than near the median.

**FIGURE 4:**
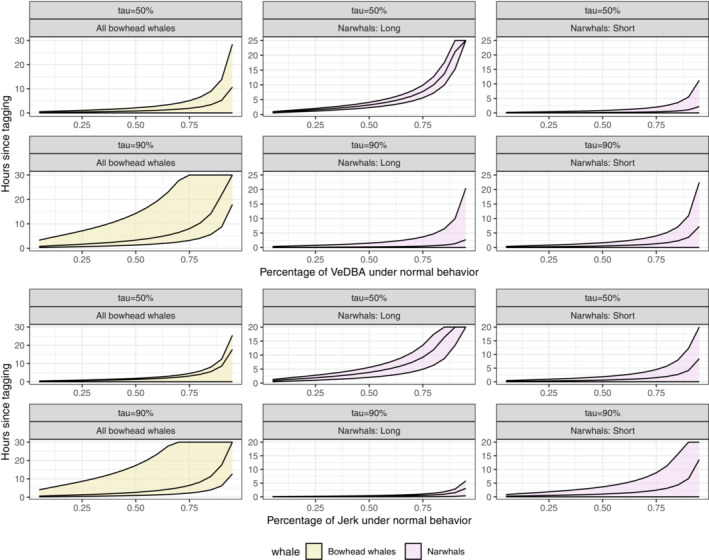
Solutions of Equation (6) for different values of δ∈[0,1[ at different quantiles (τ=0.5 and τ=0.9) along with 95% confidence intervals, when the relevant metric is jerk (lower rows) and VeDBA (upper rows). Narwhals in the middle and right columns, when handling time is long (N=12; middle) and short (N=6; right), and bowhead whales in the left column.

The aim of the model is to predict a change point in time, where the nonconstant part transfers to the constant‐effect range. Solving equation (6) for different values of δ_,_ we obtain Figure 4, which plots the time from release against the percentage of VeDBA/jerk under normal behavior ($\left(1-\delta \right)\times 100\%$). The narwhals, irrespective of handling time, quickly recovered in the tail of their distribution (τ=0.9). In contrast, in the central part of the distribution (τ=0.5), the effect of handling time had a visible effect. For narwhals with short handling times, there was a rapid recovery, whereas narwhals with long handling times was slower to recover. To make this more precise, we defined a threshold for normal behavior by setting the target percentage in Equation (6) to δ=0.25, and solved for the corresponding time. The results are summarized in Table 2. In order to evaluate the statistical significance of any differences in recovery times between quantiles or handling groups, we conducted an ANOVA analysis using the Quantreg package (Koenker, 2022). The model suggested a significant effect from handling time on the recovery of the median behavioral response of both VeDBA (p<.0001) and jerk (p<.0001) in narwhals. We found no strong evidence that tail behavior was affected by handling time for either metric (VeDBA, p=.085; jerk, p=.120). The difference in recovery between quantiles was found to be significant for the group with long handling time (VeDBA, p<.0001; jerk, p=.0007) but nonsignificant for the group with short handling time (VeDBA, p=.279; jerk, p=.527). The expected recovery of the narwhals in the median was approximately 0–10 h and <3 h in the tail. For the bowhead whales, median recovery was fast, but much slower in the tails. The uncertainty at the τ=0.9 quantile was however substantial (Figure 4). As with the narwhals, we let δ=0.25 in Equation (6) and found that the expected recovery was <4 h in the central region and roughly 6−8 h in the tail, but with huge margins of error in the latter. The difference in recovery between the quantiles was found to be significant (VeDBA, p=.001; jerk, p=.010).

**TABLE 2:** Results of quantile regression (QR) proposed in this paper and Mean Regression (MR) proposed previously (Shuert et al., 2021) for narwhals (NW) and bowhead whales (BW). In the QR approach, we define the return time $t_{R}$ (in hours) to be that instant in time, where the effect of handling and tagging is less than 25% different from normal behavior. In the MR approach, the return time $t_{R}$ is that instant in time where the deviation from the long‐time average is zero for the first time. Originally, $t_{R}$ *lower* is reported as the return time in Shuert et al. (2021).

Species	Method	Handlingtime	Quantile τ	Metric	$t_{R}$	$t_{R}$ lower	$t_{R}$ upper
Narwhal	QR	Long (*N* = 12)	50%	VeDBA	7.7	5.6	9.9
	QR		90%	VeDBA	0.5	0	3.7
	MR		‐	VeDBA	34	7	37
	QR		50%	jerk	9.2	4.9	13.5
	QR		90%	jerk	0.5	0.1	1.0
	MR		‐	jerk	7	7	37
	QR	Short (*N* = 6)	50%	VeDBA	0.4	0	2.0
	QR		90%	VeDBA	1.3	0	4.0
	MR		‐	VeDBA	17	12	64
	QR		50%	jerk	1.5	0	4.5
	QR		90%	jerk	2.4	0	8.8
	MR		‐	jerk	15	4	32
Bowhead whale	QR	All (*N* = 4)	50%	VeDBA	1.9	0	5.0
	QR		90%	VeDBA	8.0	3.1	34.4
	MR		‐	VeDBA	3	0	>20
	QR		50%	jerk	3.2	0	4.5
	QR		90%	jerk	6.4	2.3	41.6
	MR		‐	jerk	3	0	18

There is no standard practice in validating the QR model performance. A pseudo‐$R^{2}$ (denoted $R^{1}$) has been suggested as a local measure of goodness of fit, comparing models in terms of a weighted sum of absolute residuals (Koenker & Machado, 1999), but with the same deficiencies as the usual $R^{2}$. In contrast to the MR framework where the response has a specified distribution and homoscedasticity is assumed, the QR framework imposes no distributional form on the response, and the variance need not be constant. The only assumption is linearity between the response and covariates, which is easily checked by scatter plots conditioned on the location (median or 90% quantile; see Appendix S1) and independence of observations. The presence of serial correlation in the data is visible in Figure 3, which questions the validity of the last assumption, possibly leading to biased model estimates. We will return to this issue in the discussion.

### 3.2 Mean‐based regression

For comparison, we mirrored the MR framework from Shuert et al. (2021), where the quantity of interest is the difference between hourly measurements and the long‐time hourly average. The time of recovery was taken as the time when the difference between the long‐time average and hourly values was no longer significantly different from zero. For the narwhals in the present study, this difference was modeled as a generalized additive model (GAM) implemented in the mcgv R package (Wood, 2022) with time entering as a thin plate smoothing spline varying with handling time similar to one of the top‐scoring models (Table 2 in Shuert et al. (2021)). For the bowhead whales, the difference was modeled as a GAM with time entering as a thin plate smoothing spline, and no other varying or parametric terms. The return times are summarized in Table 2 along with the QR method estimates.

The results from the MR analysis were similar to the QR results at the 50% quantile for narwhals with long handling times, however, with much wider confidence bands. This difference in uncertainty was most pronounced at the upper limits where the MR analyses were more conservative than QR. For narwhals with short handling times, MR estimates were much larger than QR estimates. This is probably due to the mean being more sensitive to outliers than the median.

For the depth profiles, a direct comparison was not possible since our method involved the pair target depth and dive duration simultaneously. Nevertheless, to get some indication of the rate of recovery as predicted by MR, we applied the model to target depth. For the group of narwhals, we found a MR recovery slightly below what was predicted by the RE approach (>20 h) in the group with long handling times. The recovery was more than three times slower (>32 h) in the group with short handling times as compared to RE.

For the bowhead whales, there was no substantial difference between the methods, and both QR (in the 90% quantile) and MR shared large margins of error. Importantly, mean and median values are not directly comparable for skewed distributions such as these. The median is generally preferable and more robust for such data. The prediction of MR used on target depth and the RE method also gave similar results (<9 h).

### 3.3 Recovery of diving behavior for N=20 narwhals and N=3 bowhead whales

RE between hourly distributions of dive duration and the baseline distribution was computed for shallow, medium, and deep dives. Because BW4 only had 8 h of logged data, it was removed from the analysis, since it would not be represented in the normal region ($t\geq t_{N}=10$ h) and would potentially bias the results due to the very low sample size. Thus, we have N=3 bowhead whales for this analysis.

**FIGURE 5:**
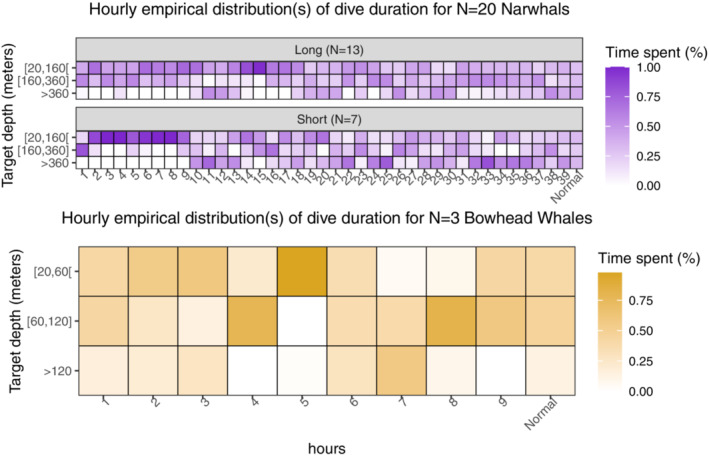
Heat map of hourly distributions of dive duration for narwhals (top) and bowhead whales (bottom) partitioned into three intervals of target depth (shallow dives, medium dives, and deep dives). Normal behavior is assumed at $t_{N}=40$ h for narwhals and $t_{N}=10$ h for bowhead whales. In this region, the baseline distribution is calculated as a weighted average over all hourly distributions.

Figure 5 shows the hourly distributions of dive durations. There is a clear discrepancy between the early distributions and the later distributions for the narwhals. Shallow dives occurred immediately after release, while deeper diving depths were attained after roughly 10 h. It is, however, hard to judge at what instant of time a change point occurs. For the bowhead whales, there are no obvious changes in the patterns, which suggests either a quick recovery or a negligible response.

**FIGURE 6:**
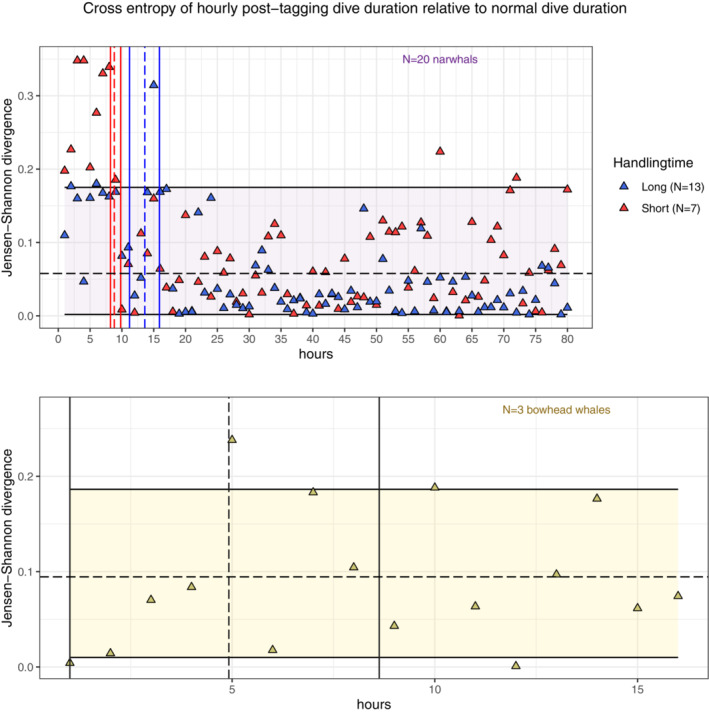
Hourly values of Jensen–Shannon RE (triangles) for narwhals (top) and bowhead whales (bottom) along with a 95% confidence interval (denoted region of recovery; horizontal band) for the expected Jensen–Shannon RE in the a priori normal region (narwhals: t≥40, bowhead whales: t≥10). The mean return times (dashed vertical lines) and confidence intervals (solid vertical lines) are computed using segmented regression on block bootstrap samples of transformed RE (with labels +1 when inside the region of recovery, and −1 when outside) with (disjoint) blocks of 2 and 5 h for bowhead whales and narwhals, respectively.

Following the procedure described in Section 2.4, we obtain Figure 6, from which a decreasing trend in the RE for both groups of narwhals is visible. The quality of the RoR can be assessed by making a pairwise comparison of the RoR's for each narwhal handling group. We compute the RoR in each subgroup of narwhals and find [0.001, 0.203] (unconditioned), [0.001, 0.062] (long handling time) and [0.001, 0.209] (short handling time). The observed difference between the RoR's of long and short handling times indicates the presence of an individual effect, which makes it difficult to conclude whether the observed difference in recovery is an artifact of handling time, is due to individual differences or—most likely—a combination of both. With such proviso, our model predicts a significantly different time of recovery between the groups of narwhals. A 95% confidence interval based on block bootstrapping is found to be [13.6, 15.9] h and [8.2, 9.8] h for narwhals with long and short handling times, respectively. Figure 6 shows that the RE of both groups of narwhals share a temporal trend up until the normal region (t>40) from where they diverge, giving rise to the different RoR's. This suggests that variability within groups is connected to the individuals, not entirely reflecting population behavior.

For the bowhead whales, the small sample size (N=3) introduces high uncertainty and potentially a huge individual bias, making the results less general. Using block bootstrapping and segmented regression, we found that the confidence interval for time of recovery is roughly [0, 8.6] h. Visually, however, we observe only few hourly entropies falling outside the RoR in a temporal disorganized fashion, which suggests a quick rate of recovery (Figure 6).

For both narwhals and bowhead whales, we observe wide RoRs, which indicates one or more of the following: (i) there is substantial hourly variation in the natural diving profiles; (ii) there are too few animals to obtain a good RoR population estimate; or (iii) the normal region is too small to capture typical behavior.

## 4 DISCUSSION

With the rapid development of biologging devices, huge streams of movement data from a variety of animals are now obtainable for researchers within ecology and related fields. Analyzing high‐resolution telemetry data and establishing novel methods that generalize well to a large class of aquatic animals, while also maintaining model transparency and observing the complexity of the sampled data, is a popular research area (Patterson et al., 2017). Establishing methods to assess recovery following any capture and tagging protocol is of particular importance, namely to avoid mixing data representative of normal behavior with data contaminated by the effect of tagging (Blackwell et al., 2018; Lennox et al., 2017; Shuert et al., 2021). In this paper, we have presented two recovery estimation techniques that allow for subdivision of animals based on selected covariates, and have applied the methods to data from N=20 narwhals and N=4 bowhead whales tagged in Greenland with Acousonde™ tags.

Measures of energy expenditure and activity (VeDBA and jerk) were derived from accelerometer data and analyzed using QR with an individual offset and a slope dependent on a predefined collection of covariates in the linear predictor function. One of the strengths of QR, as opposed to ordinary regression, is the incorporation of heterogeneous variance over different locations of the distribution, as well as possible shifts in the mean estimand (Cade & Noon, 2003).

Using QR and focusing on the median and the 90% quantile, we found evidence of such heterogeneity for the East Greenland narwhals and likewise for the West Greenland bowhead whales, which displayed a significant difference between quantiles, despite the low sample size and limited records. For the narwhals, we found a variable return time to normal behavior, depending on both handling time and the location of the distribution. Time of recovery for narwhals was in general achieved in much less than a day (<10 h) as also indicated in a previous study (Shuert et al., 2021), but with a significant difference between the median (>4.9 h) and 90%‐quantile (<3.7 h) in the group with long handling times. Our model predicted quick recovery of the bowhead whales in the median (∼2–3 h), but slower recovery in the tail of the distribution (∼6–8 h). The tail estimates were however accompanied by huge uncertainties. Moreover, the presence of serial correlation might lead to underestimation of standard errors. We attempted to minimize the degree of serial correlation by aggregating measurements into hourly observations. Inherent to QR is also the restriction of the response to various locations of the distribution, which in itself might reduce any existing serial correlation compared to the complete set of observations.

Complicated relationships between predictor variables and response often arise in ecology. One could therefore easily imagine QR as one of the future standard toolbox methods for ecologists and biologists alike, when analyzing biologging data (Cade & Noon, 2003; Koenker & Machado, 1999). For cetaceans in particular, locomotive metrics (such as VeDBA and jerk) are expected to be closely linked with activities, giving rise to location‐scale distributions. For example, we expect energy expenditure to be lower and have a smaller variance when a whale is resting, and to be higher and more variable during foraging.

For both species, we compared our QR results to MR as proposed by Shuert et al. (2021). The two methods agreed for central recovery of VeDBA and jerk for narwhals with long handling times and bowhead whales, but they did not agree for narwhals with short handling times. We predicted that mean and median values might not be directly comparable as central markers when extreme observations are widely present (see Figure 2), or when the data display unequal recovery trends at different distributional locations. The late recovery and huge margins of error of the mean values (see Table 2) might be a consequence of the fact that the mean expresses overall recovery, in contrast to the quantiles, which reveal differences in activity levels based on the location of the distribution. When we focus the regression on different distributional locations, the observations become less scattered. By furthermore forcing a monotonic pattern as proposed in the QR model, we obtain more precise estimates at both the median and 90% quantile.

In addition to energy and activity profiles, diving behavior was addressed using the concept of relative entropy (RE), also called divergence, by comparing hourly distributions of dive duration partitioned into three intervals of target depth (shallow, medium, and deep dives) to a long‐term average distribution. The component pair (target depth and dive duration) together produced a coarse profile of the diving behavior, but did not capture the finer details of the dives.

Unlike QR, the RE approach contained several steps toward estimating resumption of normal diving behavior. A conservative guess at a normal region was selected and from that we constructed a 95% confidence interval (RoR) for the RE between hourly distributions of dive duration and distribution within the normal region. Estimated RE outside and inside the RoR was assigned a value of −1 and +1, respectively. We took the return time to be the expected breakpoint (over block bootstrap samples, using segmented regression) of the cumulative sums of the assigned values.

Applied to the study animals, our results show that the recovery of diving behavior took significantly longer than that of the accelerometer metrics (VeDBA and jerk) for both groups of narwhals, a finding shared by Shuert et al. (2021). This finding may be due to the close dependency of the diving behavior metrics (e.g., target depth) on whether or not the whales were foraging and is, therefore, not exclusively related to the tagging procedure. After release from capture, narwhals generally abstain from echolocating (thus presumably feeding) for hours to days (Blackwell et al., 2018; Shuert et al., 2021). Instead, they move away rapidly (Heide‐Jørgensen et al., 2021), using relatively shallow dives. In addition to these behavioral factors, the area in which the whales were tagged is not a known or preferred foraging area, so deep dives would not be expected there. Our model also suggests a significant difference due to handling time, but the presence of individual bias resulting from few animals could be an important contributing factor in addition to handling time. For the bowhead whales, the results also suggest a slower recovery in diving profiles compared with the accelerometer results. This is, however, to be expected with the limited number of individuals.

In the RE‐analysis, we used several control parameters including the threshold for the normal region $t_{N}$, a temporal upper limit T and the block size in bootstrapping. We required T to be small enough that hourly distributions in the late temporal stage are not substantially different due to fewer monitored whales. Assigning T to an early drop point is particularly important when the animal sample size is small, as the hourly distribution will likely change significantly following the removal of a few animals. This could lead to multiple occurrences of the label −1 in the later temporal stage due to unintended shifts in hourly distribution, potentially resulting in overestimation of the break point. $t_{N}$ on the contrary needs to be large enough, to allow for the possibility of a potentially long recovery time and proper estimation of the long‐term reference distribution Q. While $t_{N}$ indicates the threshold for a normal region, and thereby the time at which the calculation of the reference distribution is initiated, the procedure in principle allows for later recovery times. For example, if we underestimate the recovery $t_{N}$, and the subsequent hourly distributions (i.e., $P_{t}$ with $t>t_{N}$) are very different from the reference distribution Q. In such a situation, the corresponding RE measurements will potentially exit the RoR and the segmented regression used on the binary labels could potentially identify a breakpoint greater than $t_{N}$. These requirements put some restrictions on the choice of $t_{N}$ and T, which must be carefully fixed based on data availability. Regarding block size, we are aware that the standard error of the breakpoints is by construction intrinsically connected to the block bootstrap procedure, in which we used blocks with 5 h and 2 h temporal widths for narwhals and bowhead whales, respectively. Making the block width too small (relative to how the data cluster) decreases the variability of the bootstrap samples, and when too large, the serial correlation of the data are not properly accounted for. Another possibility is to make the width variable and automated, but the choice should ideally depend on the data‐generating process and the statistic (the breakpoint) to be estimated (Hall et al., 1995). Determining optimal block width will be a natural next step to properly account for the variability. A final subtlety worth mentioning is the use of hourly distributions, even though the method could easily be expanded to using temporal intervals of length relevant to the species being investigated. One could even hypothesize that longer intervals shrink the bias resulting from a small sample size, as (groups of) animals might be more similar when observed over longer time periods; at least for diving behavior. Despite these challenges, we believe the RE method to be a general, transparent, and statistically sound way of characterizing the diving profile and its return to normal behavior following tagging.

The findings of this study indicate that recovery from tagging is potentially influenced by various factors, such as the metric being analyzed (e.g., depth or acceleration), the duration of tag deployment, and potential differences in recovery across activities. This means that ecologists conducting behavioral studies of marine animals must carefully consider how much data to discard, depending on the research question. For instance, researchers tracking several physiological or activity metrics using multisensor biologgers would have to measure the recovery within each metric, and select the maximum time of recovery as the cutoff point for data trimming to obtain a natural and untainted animal profile. The most striking effect identified in the narwhal case study is the impact of handling time. Even an average difference of half an hour between groups can result in substantial differences in recovery times. For example, this small deviation in handling time resulted in an expected difference of 5 h in recovery time for the diving profile described by target depth and dive duration (Table 3) and an expected difference of approximately 7 h in the median in terms of fine‐scale movement as measured by VeDBA and jerk (Table 2). This emphasizes the need for reducing handling time, as even a few minutes can potentially impact recovery substantially. It is expected that the tagging procedure also plays a central role in the behavioral response of the species. For example, the narwhals were captured with set nets and tagged near shore, whereas the bowhead whales were pursued and tagged at sea. The reactions to being pursued and/or captured might be markedly different, which also calls for a standard practice (Walker et al., 2012) if the results of baseline studies are to be applicable in general.

**TABLE 3:** Results of RE analysis. Estimated return times $t_{R}$ (breakpoints) in hours for narwhals (NW) and bowhead whales (BW) using segmented regression on block (narwhals, width = 5 h; bowhead whales, width = 2 h) bootstrap samples of transformed Jensen‐Shannon RE measurements assigned a value of negative one when inside, and positive one when outside the region of recovery.

Whale	Handling time	$t_{R}$	$t_{R}$ lower	$t_{R}$ upper
NW	Long	13.6	11.2	15.9
NW	Short	8.8	8.2	9.8
BW	‐	4.9	<1.0	8.6

While the methods are simple to implement and applicable over a broad range of marine species, there are certain pitfalls to consider. Here, we briefly mention a few. On statistical grounds, there is the matter of variability. Movement ecology of aquatic animals has a complex composition (Patterson et al., 2017) where unequal within‐individual variances arise due to regular transitions between activities (again justifying QR) such as resting, foraging and so forth, but also with substantial between‐individual variance (Heide‐Jørgensen & Dietz, 2011; Laidre et al., 2002). Mapping out traits that classify animals into groups with minimal between‐individual variance with respect to a set of behavioral criteria should be considered, for a fairer comparison between small groups of animals. In this paper, we analyzed N=20 narwhals and further divided them into two groups based on handling time (Table 1) with low within‐group variation and high between group variation. But are the groups even comparable? For example, we found that the narwhals with short handling times were on average larger than those in the group with long handling times. As a consequence, any difference in results between groups could potentially be influenced by size or other factors. While it is recognized that randomization over many animals has the potential of removing confounding factors, in practice, we only have a relatively small sample, which stresses the importance of mapping out traits that influence recovery.

In our analyses, we have assumed that the chosen metrics are well‐suited for explaining the target behavior. For example, we relied on previous studies (Shuert et al., 2021; Wilson et al., 2020) that suggested DBA (Dynamic Body Acceleration) as a prudent way of estimating energy expenditure. Meanwhile, other studies suggest only a weak correlation between DBA and the rate of oxygen consumption, if not also conditioned on different activities (Jeanniard‐du Dot et al., 2017; Ladds et al., 2017; Martín López et al., 2022; Tervo, Ditlevsen, et al., 2021). While overall DBA still relates to activity, the interpretation as a measure of energy expenditure might not be valid. Another subtlety on the usefulness of DBA is that its derivation presupposes that one can filter out the component of orientation (ascribed to gravity). If this is not the case, then dynamic acceleration, hence also DBA, might contain a sizeable static component. This effect seems to be predominantly associated with larger animals, and negligible for smaller ones. In that light, the interpretability of VeDBA as a measure of energy expenditure for the large bowhead whales used in this study might be flawed. The narwhals used are borderline cases (Martín López et al., 2022).

In the present paper, we have not given thorough attention to the validity of the metrics, but instead focused on how *meaningful* metrics derived from high temporal resolution accelerometer and depth data can be used in conjunction with our proposed methods to assess the time of recovery following tagging. The results of this paper were, however, repeated with Overall Dynamic Body Acceleration (ODBA) replacing VeDBA, defined as $\text{ODBA}=|{\mathrm{DA}}_{x}|+|{\mathrm{DA}}_{y}|+|{\mathrm{DA}}_{z}|$. Several studies have found that these two metrics are not significantly different over a wide range of activities in predicting the rate of oxygen consumption (Ladds et al., 2017; Wilson et al., 2020). We also found no deviation in results using either metric (see Appendix S1). In contrast to DBA, we believe that the designated metrics for diving behavior (target depth and dive duration) come with an appealing and natural interpretation. Our hope is that the presented techniques provide a flexible toolbox to estimate recovery from the effect of tagging, by allowing researchers to include a set of relevant covariates relating to the study animals. We have discussed potential deficiencies and highlighted areas where tuning to the specific study species is required, allowing researchers to calibrate the methods where needed.

## AUTHOR CONTRIBUTIONS

**Lars Reiter Nielsen:** Conceptualization (equal); formal analysis (lead); investigation (equal); methodology (equal); visualization (equal); writing – original draft (lead); writing – review and editing (equal). **Outi Tervo:** Conceptualization (equal); investigation (equal); project administration (equal); supervision (equal); visualization (equal); writing – review and editing (equal). **Susanna B Blackwell:** Data curation (equal); funding acquisition (equal); validation (equal); writing – review and editing (equal). **Mads Peter Heide‐Jørgensen:** Data curation (equal); funding acquisition (equal); investigation (equal); resources (equal); writing – review and editing (equal). **Susanne Ditlevsen:** Conceptualization (equal); funding acquisition (equal); investigation (equal); methodology (equal); project administration (equal); supervision (equal); writing – review and editing (equal).

## FUNDING INFORMATION

Novo Nordisk Foundation (NNF20OC0062958); Independent Research Fund Denmark | Natural Sciences (9040‐00215B); Carlsberg Foundation, CF14‐0169; Danish Cooperation for the Environment in the Arctic (DANCEA), 2013_01_0289; the Bureau of Ocean Energy Management, USA (Contract M12PC00005 for Acousonde™ instruments used on bowhead whales).

## CONFLICT OF INTEREST STATEMENT

SBB is employed by Greeneridge Sciences, Inc. There are no patents to declare, but the Acousonde™ tag used in this study is manufactured by Acoustimetrics, a brand of Greeneridge Sciences, Inc.

### OPEN RESEARCH BADGES

This article has earned an Open Data badge for making publicly available the digitally‐shareable data necessary to reproduce the reported results. The data is available at [http://doi.org/10.5281/zenodo.7334175].

## Supporting information


Appendix S1.

## Data Availability

Rmarkdown files and R scripts used to analyze the archival datasets are available at GitHub account *LaReiter* under http://doi.org/10.5281/zenodo.7334175, where the preprocessed datasets are also found.
