# Supplementary material for: Using quantile regression and relative entropy to assess the period of anomalous behavior of marine mammals following tagging
Source: Ecol Evol. 2023 Apr 10;13(4):e9967. doi: 10.1002/ece3.9967 (PMC10085821; doi:10.1002/ece3.9967)
Supplement: Supplementary file 1 — Appendix S1. [file ECE3-13-e9967-s001.pdf]

# Supplementary material for Using Quantile regression and Relative entropy to assess the period of anomalous behaviour of marine mammals following tagging

Lars R. Nielsen Outi M. Tervo Mads Peter Heide-Jørgensen
Susanna B. Blackwell Susanne Ditlevsen

18 november 2022

## Contents

Create artificial data . . . . . . . . . . . . . . . . . . . . . . . . . . . . . . . . . . . . . . . . . . . 1
Method 1: Quantile Regression . . . . . . . . . . . . . . . . . . . . . . . . . . . . . . . . . . . . . . . 5
Method 2: Jensen-Shannon Divergence . . . . . . . . . . . . . . . . . . . . . . . . . . . . . . . . . . 9
Supplementary figures (on actual datasets) . . . . . . . . . . . . . . . . . . . . . . . . . . . . . . . 21

In this note, we will show how implementation of the methods "Quantile Regression" and "Jensen-Shannon Divergence" are achieved in the free software R for statistical computing (R Core Team, 2022). Using simulated data, we will then recreate similar figures as those in the article and also show how supplementary figures (not present in the article) are obtained. Finally, we will present all supplementary figures on the **actual datasets** of the study animals. The "Mean-based Regression" code is available as a script "ReproduceCanada-NW.R" and should be easy to follow. For details on the implementation, see section "Applying mean based regression" in the main article. All datasets as well as original scripts are available here https://github.com/LaReiter/TaggingEffects

We use the following R packages:

```
library(ggplot2)   ## Grammar of graphics
library(reshape2)  ## Reshaping data frames
library(dplyr)     ## dplyr functionality
library(data.table) ## data tables and data tables functionality
library(readr)     ## read csv
library(gridExtra) ## graphics
library(xtable)    ## LaTeX formatting of tables
library(splines)   ## splines
library(grid)      ## graphics
library(quantreg)  ## quantile regression
library(segmented) ## segmented regression
theme_set(theme_bw()) ## set black and white plots
```

## Create artificial data

### Functions for simulation

We will first introduce the functions used to generate artificial data for both finescale metrics (such as VeDBA and Jerk) as well as depth data. The below procedures ignores serial correlation, and focus solely on reproducing the general trend visible in the datasets of the research paper.

```r
set.seed(111) # for reproducibility

##################################################################################
## Simulate 'finescale' metric (eg. VeDBA, Jerk) - assume exponential decay and ##
## with Gamma Noise. The goal is to present the procedure, so the exact         ##
## distributional form is immaterial                                            ##
##################################################################################

simFineScale <- function(Time,RecTime){
  # time in hours, median recovery time, upper quantile recovery time

  TimeVec <- 1:(Time*60) # time in minutes

  # add variation to recovery time
  recoveryOffset <- sample(-5:5,1) # variation in recovery

  # recovery (change) points
  changepoint <- (RecTime+recoveryOffset)

  # add gamma noise with scale/rate exponential decaying parameters
  shapeAndScale <- 2*exp(-rep(1:Time,each=60)/changepoint)
  noiseg <- rgamma(TimeVec,shape=shapeAndScale,
                    scale=shapeAndScale)
  # and add normal noise with uniformly distributed std deviation
  noisen <- rnorm(1:(Time*60),0,runif(1:(Time*60)))

  # generate observations y and offset to y>0 (data assumed positive)
  y <- noiseg+noisen
  y <- y + abs(min(y))+1e-1

  return(y)

}



###############################################################################
## Simulate Depth metric  - assume a random walk with three states: surface, ##
## foraging, deep diving and ascending where each state has a probability    ##
## which is dependent on whether the whale has recovered or not              ##
###############################################################################

########################
## Auxiliary function ##
########################
getDepth <- function(x){
  ifelse(x=="Sh",return(sample(40:120,1)),
         ifelse(x=="M",return(sample(120:300,1)),
                return(sample(300:600,1))))
}

simDepth <- function(Time,RecTime){
  # Time in hours, Recovery lower limit, Recovery upper limit
```

```r
# Time of recovery in hours with variation
TimeOfRecovery <- sample(max(0,RecTime-5):((RecTime+5)),1)

states <- c("S","Sh","M","D","A") # Surface,Shallow,Medium,Deep diving,Ascending
curState <- states[5] # Set ascending (start in a dive; see Depth section later)

# check if diving
divingDummy <- TRUE # start in a dive

baseTransitionProb <- 0.99 # base transition probability from surface to diving
transitionProb <- 0.99 # variable transition probability

# transition probabilities form Surface (or bottom of dive) to ...
statesProb <- c(0.9,0.05,0.05,0.3) # Shallow,Medium, Deep diving and Ascending
statesProbRec <- c(0.1,0.5,0.4,0.3) # (...) after recovery

# speed (in units)
speed <- c(4,8,12,6) # Shallow,Medium,Deep Diving and Ascending speed
speedSigma <- c(3,3,3,3) # Standard deviation

depthVec <- rep(0,Time*60) # vector of depths
depthVec[1] <- getDepth("Sh") # initiate at some Shallow depth

for(t in 2:(Time*60)){ # time in minutes

  # recovery transition probabilities
  if(t>TimeOfRecovery*60){statesProb <- statesProbRec}

  # check if we are at surface level
  if(curState=="S"){
    divingDummy <- sample(c(1,0),1,
                   prob=c(1-transitionProb,transitionProb)) # change state
    if(divingDummy){ # now diving
      curState <- sample(states[-c(1,5)],1,prob=statesProb[-4])
      targetDepth <- getDepth(curState) # get random target depth
      transitionProb <- baseTransitionProb # reset surface transition probability
    }
    else
    {
      depthVec[t] <- depthVec[t-1]+rnorm(1,0,3) # continue roaming
      transitionProb <- transitionProb*baseTransitionProb # increase chance of transition
    }
  }
  if(curState %in% c("Sh","M","D")){ # check if we are diving or foraging
    if(divingDummy){ # check if we have reached target depth...
      speedIdx <- ifelse(curState=="Sh",1,ifelse(curState=="M",2,3)) # get speed vector index
      depthVec[t] <- depthVec[t-1]+speed[speedIdx]+rnorm(1,0,speedSigma[speedIdx]) # update
      if(depthVec[t]>targetDepth)divingDummy <- FALSE # target depth reached
    }
    else{ #... or if we are about to ascend
      depthVec[t] <- depthVec[t-1]+rnorm(1,0,3) # continue roaming
      if(runif(1)>statesProb[4])curState<-"A" # now ascending
    }
```

```
    }
    # if we are ascending
    if(curState=="A"){
      depthVec[t] <- depthVec[t-1]-speed[4]+rnorm(1,0,speedSigma[4]) # update
      if(depthVec[t]<20)curState <- "S" # we are back at surface level (defined < 20m)
    }
  }
  # return random walk
  return(depthVec)
}
```

### Gather data into dataframe

We now use the procedures above to generate artificial data for $N = 10$ individuals, of which half has a categorical covariate with a value $A$ and the other group has value of $B$. Type A corresponds to quick recovery relative to B. The length of the observations of different individuals are of uneven size (in order to introduce weighting protocol for depth data).

```
################################################################################
## Create 10 aquatic animals distributed into 2 groups with one covariate     ##
## which affects the recovery in an exaggerated fashion. The covariate is a   ##
## 2-level factor, where factor A means the recovery is (on average) twice as ##
## fast as the recovery of the group with factor B                            ##
################################################################################


RunTime <- c(50+(0:4)*10,50+(0:4)*10) # total runtime (vector) in hours
# AVERAGE recovery times:
RecTime_Fine_A <-5 # fine recovery of category A whales in hours
RecTime_Fine_B <- 20 # fine recovery of category B whales in hours
RecTime_Depth_A <- 10 # coarse recovery of category A whales in hours
RecTime_Depth_B <- 30 # coarse recovery of category B whales in hours


# initiate dataframe
df <- data.frame(
  ind=integer(), # index of animal
  time=numeric(), # time
  fineM=double(), # fine scale metric
  depth=double(), # depth metric
  covariate=character() # factor covariate (A,B,C,...)
)

# generate data
N <- 10 # 10 individuals
for(n in 1:N){
  if(n>5){ # half has covariate=A
    df0 <- data.frame(ind=n,
                      min=1:(RunTime[n]*60),
                      fineMetric=simFineScale(RunTime[n],RecTime_Fine_A),
                      depth=simDepth(RunTime[n],RecTime_Depth_A),
                      covariate="A")
  }
  else{ # ... and half has covariate=B (half as fast)
```

```
    df0 <- data.frame(ind=n,
                      min=1:(RunTime[n]*60),
                      fineMetric=simFineScale(RunTime[n],RecTime_Fine_B),
                      depth=simDepth(RunTime[n],RecTime_Depth_B),
                      covariate="B")
  }
  df <- rbind(df,df0) # append
}
```

### Visualize

Visualising the data for a single individual, we obtain the following plots:

```
p1 <- ggplot(df[df$ind==1,],aes(min,fineMetric))+
  geom_jitter(alpha=I(0.2),color="gray0")+
  geom_smooth(color="red")+
  ylab("Fine scale metric (unitless)")
p2 <- ggplot(df[df$ind==1,],aes(min,depth))+
  geom_jitter(alpha=I(0.2),color="gray0")+
  geom_smooth(color="blue")+
  ylab("Depth (meters)")
grid.arrange(p1,p2,nrow=1,top="Simulated data for individual #1")
```

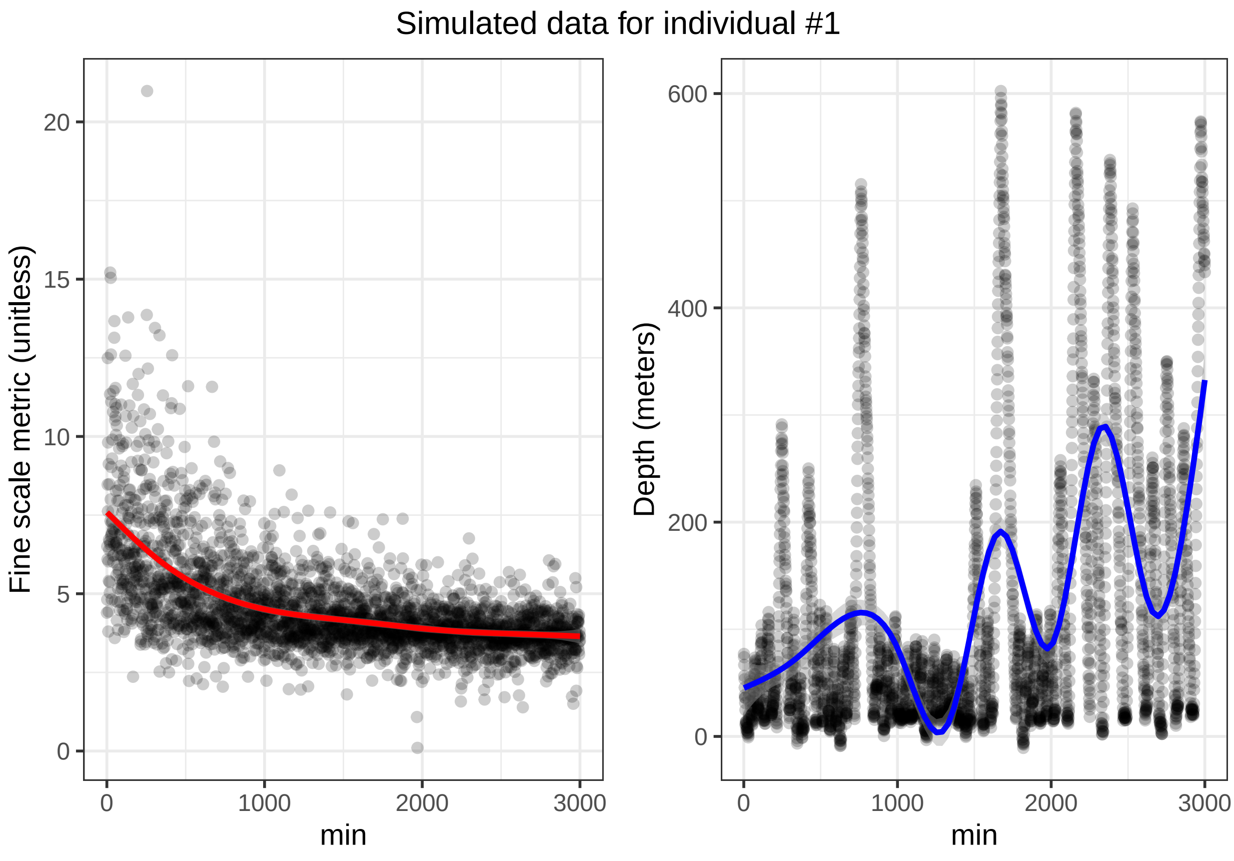


## Method 1: Quantile Regression

Here we present the method and R-implemention for Quantile Regression (QR).

There are no distributional assumptions for the response in the QR model, and we do not require homogenity of variance. We do however assume a log-linear relationship between the response and the predictor variables:

$$\log(Y^\tau)\,|\,\mathbf{X}, t = \beta_{Ind} + \alpha\left(\frac{1}{t+1}\right) \tag{1}$$

This linearity assumption can be hard to verify, since we have a cluster of points when $z \equiv \frac{1}{t+1}$ is small (when $t$ is large) and only few when $z$ is large ($t$ is small). We can however introduce a slight time lag, and disallow $z$ to be too large (and $t$ too small). Below we focus on the interval between 5 and 50 hours.

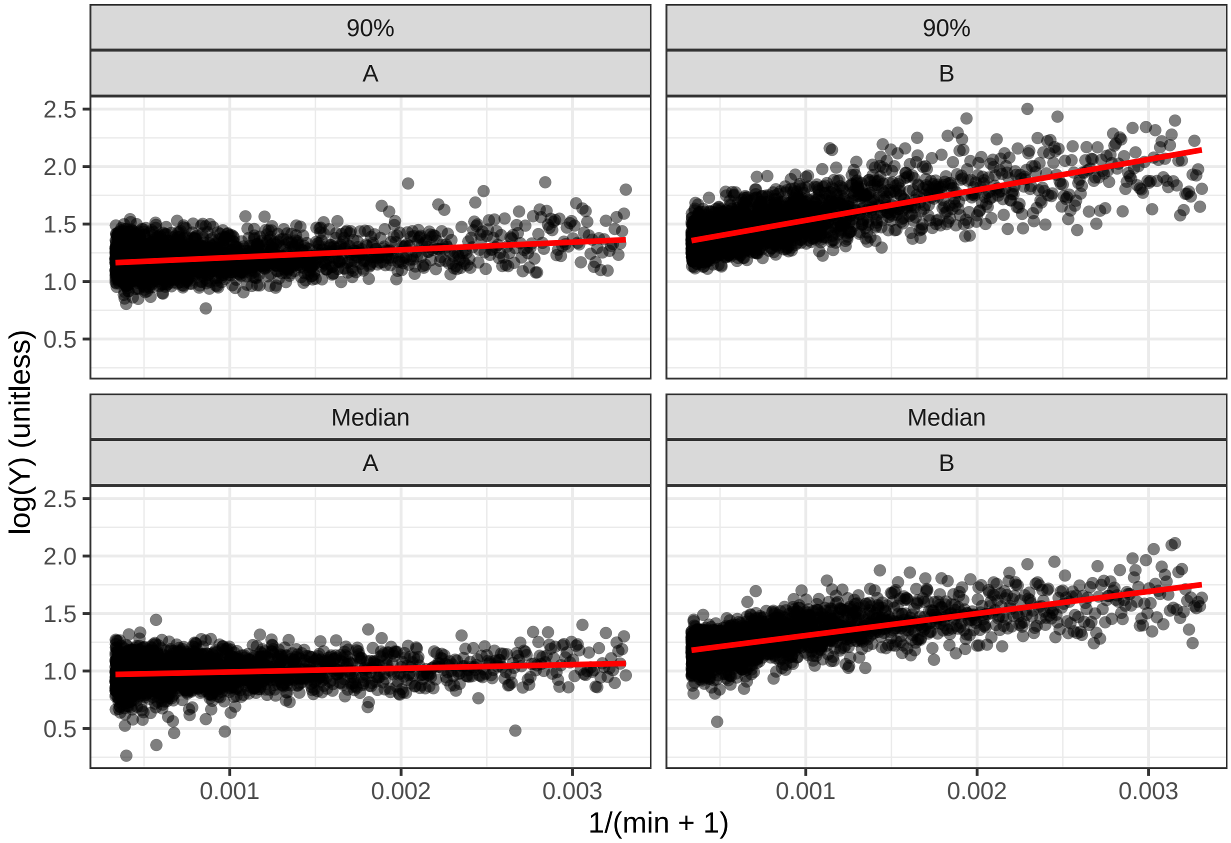


Fitting the QR model is done using the Quantreg package:

```
# QuantReg: https://cran.r-project.org/package=quantreg
library(quantreg)

# fit
formula <- log(fineMetric) ~  factor(ind)+factor(covariate):I(1/(min+1))-1
qr.model <- rq(formula, tau=c(0.5,0.9), data=df)

# predictions
df$Q90 <- exp(predict(qr.model,newdata=df))[,2] # 90% quantile
df$Q50 <- exp(predict(qr.model,newdata=df))[,1] # 50% quantile
```

The QR equation (1) gives rise to the equation

$$Y^\tau = \beta^* \exp\left(\alpha \frac{1}{t+1}\right)$$

where we can ask at what time $t$ the response changes less than some $\delta \in (0, 1)$. This is solved below

```
delta <- seq(0.05,0.95,0.05) # deltas (percentage of change)

# vector of metrics to be studied (eg. VeDBA and Jerk)
met.vec <- c("fineMetric")
# vector(s) of covariate(s) - in this case we only have one
cov.vec <- c("A","B")
# vector of quantiles - here 50% (median) and 90%
tau.vec <- c("50%","90%")

ml <- length(met.vec) # length
cl <- length(cov.vec) #...
tl <- length(tau.vec) #...
size <- ml*cl*tl # size of data.frame (see below)

# dummy dataframe
df.est <- data.frame(
  met = rep(met.vec,length.out=size), # 1st level: metric
  cov = rep(cov.vec,each=size/cl), # 2nd level: covariate
  tau = rep(rep(tau.vec,size/(ml*tl)),ml), # 3rd level: tau
  t.lower = NA, # upper limit of recovery time
  t.upper = NA, # lower limit of recovery time
  t = NA # recovery time
)

for(c in 1:cl){
  for(t in 1:tl){
      SE <- summary(qr.model)[[t]][3][[1]][N+c,2] # standard error
      est <- summary(qr.model)[[t]][3][[1]][N+c,1] # estimate
      # update dataframe:
      df.est[df.est$met=="fineMetric" & df.est$cov==cov.vec[c] & df.est$tau==tau.vec[t],c("t.lower","t.u
  }
}

# auxiliary functions
posOnly <- Vectorize(function(x) ifelse(x>0,x,0))
minOf <- Vectorize(function(x,y) min(x,y))
maxOf <- Vectorize(function(x,y) max(x,y))

reps <- 2*2*1 # covariates*quantiles*metrics
df0 <- data.frame(
  delta = rep(delta,reps), # 1st level
  t = rep(delta,reps), # 1st level
  Ut = rep(delta,reps), # 1st level
  Lt = rep(delta,reps), # 1st level
  tau = rep(tau.vec,each=((reps/tl)*length(delta))), # 2nd level
  cov = rep(rep(cov.vec,each=((reps/(cl*tl))*length(delta))),ml), # 3rd level
  met = rep(rep(met.vec,each=length(delta)),cl*tl) # 4th level
)

# CONSTRUCT dataframe for plotting (containing Time, CI of Time)
for(m in met.vec){
  for(c in cov.vec){
```

```
    for(t in tau.vec){

      e <- df.est[df.est$met==m & df.est$cov==c & df.est$tau==t,]$t
      e.l <- df.est[df.est$met==m & df.est$cov==c & df.est$tau==t,]$t.lower
      e.u <- df.est[df.est$met==m & df.est$cov==c & df.est$tau==t,]$t.upper


      # Estimates
      df0[df0$met==m & df0$cov==c & df0$tau==t,]$t <- posOnly(-(abs(e)/log(delta)))

      # CI lower and CI upper:
      if(e<0){
        df0[df0$met==m & df0$cov==c & df0$tau==t,]$Lt<- posOnly(e.u/log(delta))
        df0[df0$met==m & df0$cov==c & df0$tau==t,]$Ut<- posOnly(e.l/log(delta))
      }
      else
      {
        df0[df0$met==m & df0$cov==c & df0$tau==t,]$Ut<- posOnly(-e.u/log(delta))
        df0[df0$met==m & df0$cov==c & df0$tau==t,]$Lt<- posOnly(-e.l/log(delta))
      }
    }
  }
}
```

```
## Warning: Using `size` aesthetic for lines was deprecated in ggplot2 3.4.0.
## i Please use `linewidth` instead.
```

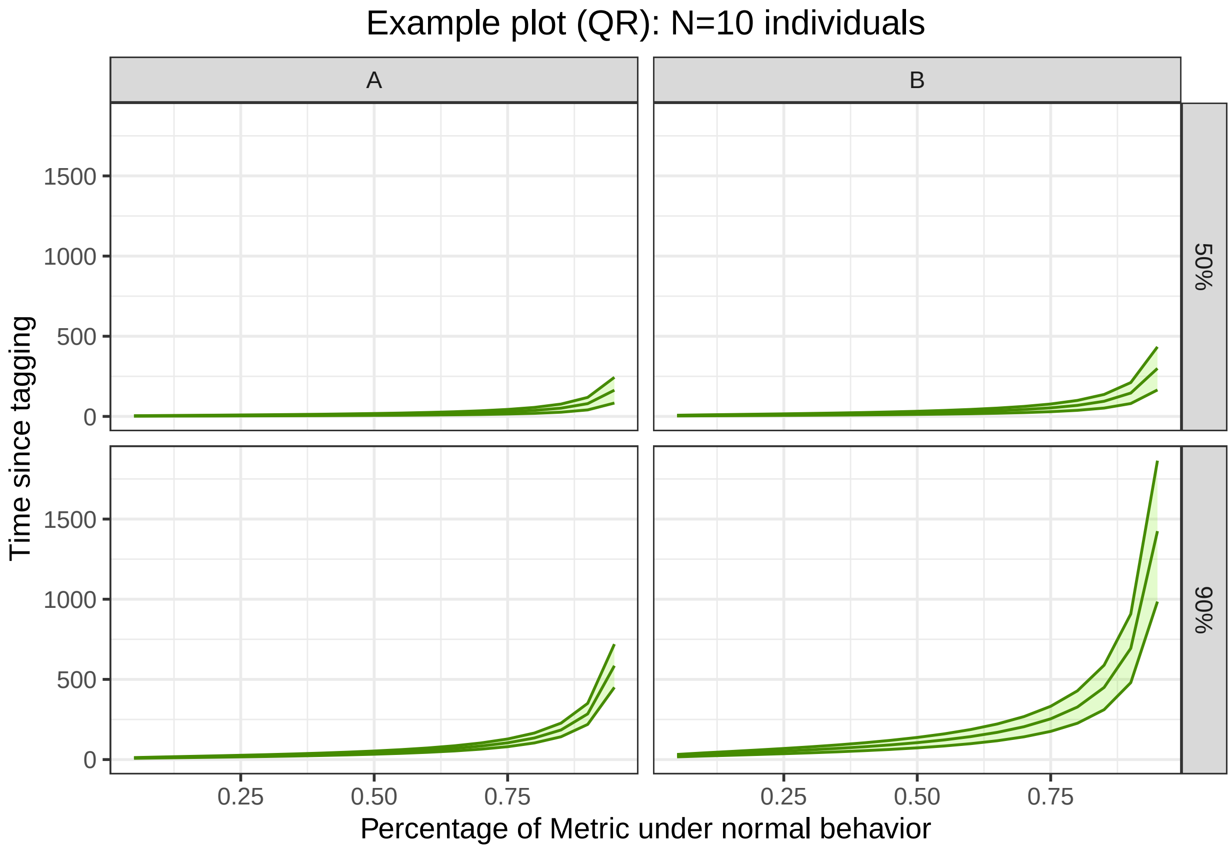

Finally we visualize the fits:

```
# visualize
ggplot(df[df$ind %in% c(2,7) & df$min>60,],aes(x=min))+
  geom_point(aes(y=fineMetric),alpha=I(0.2))+
  geom_line(aes(y=Q50),size=0.5,colour="blue")+
  geom_line(aes(y=Q90),size=0.5,colour="red")+
  xlab("")+
  ylab("Fine scale metric (unitless)")+
  facet_wrap(ind~.,scales="free")
```

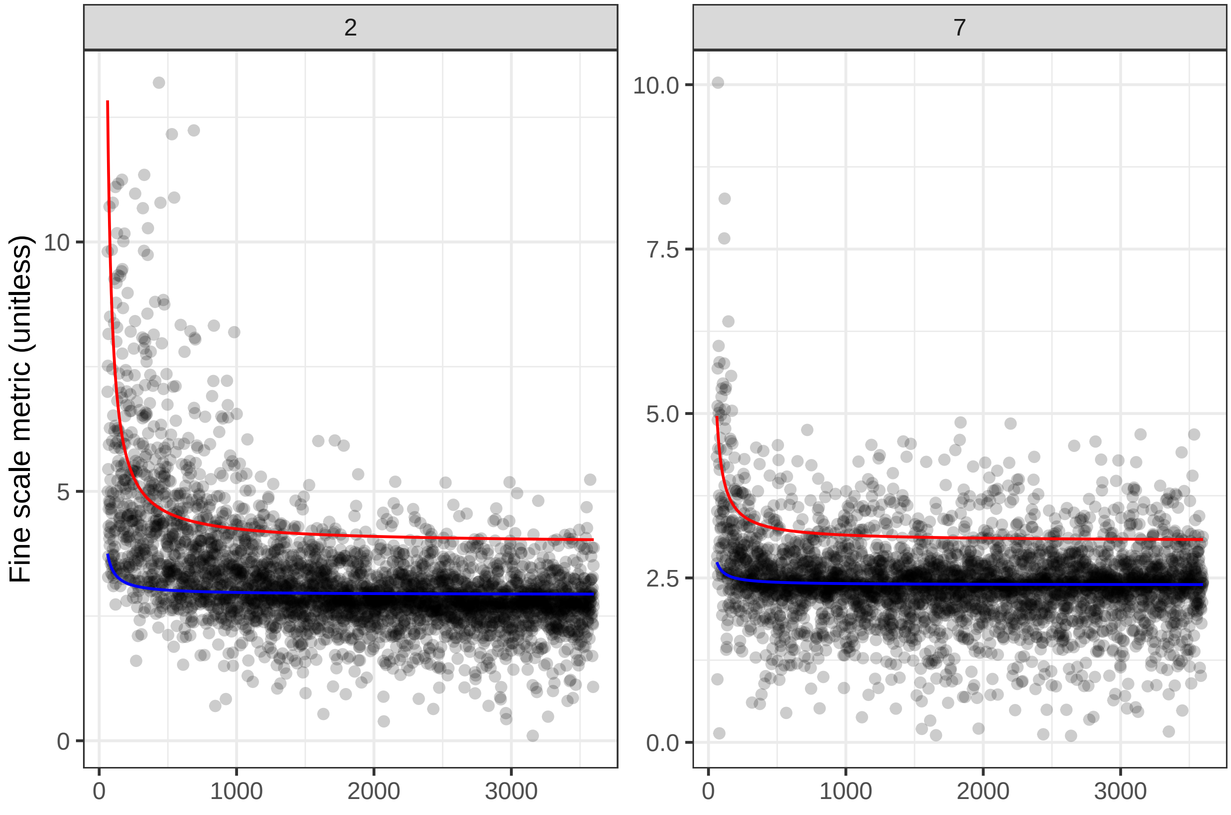


## Method 2: Jensen-Shannon Divergence

In this section, we present how the Depth Diagrams and Jensen-Shannon Divergence graphs are constructed. We also present the implementation of the Bootstrap procedure (for achieving confidence intervals relating to time of recovery) and visualize bootstrap samples. Notice that the low overall variation in the bootstrap samples does not imply low variation in the change point estimates. Long identical stretches, with even minor variation around selected time points, will still result in notable shifts in the breakpoints of the relevant bootstraps samples.

### Derive Target Depth and Dive duration from Depth data

```
# divide depths into segments of 20 meters
depthWidth <- 20

# modify dataframe
df<- mutate(df,
```

```
            depthlvl = floor(depth/depthWidth),  # depth level = interval of 20
            TD = rep(0,nrow(df)), # target depth
            TDD = rep(0,nrow(df))) # dive duration

##################################################
## Construct 'Target depth' and 'Dive duration' ##
##################################################

# Remove consecutive dublicates of depthlvl.
# X contains two elements (values = Depthlvl)
# and (lengths = Occurences of values)
X <- rle(df$depthlvl)

maxdepthlvl <- max(X$values) # maximum depth lvl
surfaceidx <- which(X$values %in% c(0)) # all surface indices

# The dummy variables depend upon the TAIL and FRONT (if we start/end at surface)
front.idx <- X$values[1]>0
tail.idx <- tail(X$values,1)>0
dummy.L <- 2*length(surfaceidx)+1 # number of dives
if(tail.idx != front.idx ){dummy.L <- 2*length(surfaceidx)} # adjust

# ILLUSTRATION of the principle above on the 4 lines above:
#   0 1 0 1 0   # Start: Surface, End: Surface  3 dives
#   1 0 1 0 1   # Start: Dive, End: Dive     3 dives
#   0 1 0 1 -   # Start: Surface, End: Dive   2 dives
#   1 0 1 0 -   # Start: Dive, End: Surface    2 dives
# Here 0 represents "surface" and 1 represents "dive".
# Hence if we are at surface at the start and dive in
# the end or vice versa, the no. dives is only 2*"surface appearances"

dummy1 <- rep(0,dummy.L) # initiate dummy for TD (Target Depth)
dummy2 <-rep(0,dummy.L) # initiate dummy for TDD (Dive Duration)


#####################################
## Dummy variable for Target Depth ##
#####################################

if (front.idx == 0){ # if we start at surface
  dummy1[1] <- 0
  for(i in 2:(length(surfaceidx))){
    # get max depth between two consecutive surface lvls
    dummy1[2*(i-1)] <- max(X$values[surfaceidx[i-1]:surfaceidx[i]])
  }
}

if (front.idx == 1){ # if we start in a dive
  dummy1[1] <- max(X$values[1:surfaceidx[1]])
  for(i in 2:(length(surfaceidx))){
    # get max depth between two consecutive surface lvls
    dummy1[2*i-1] <- max(X$values[surfaceidx[i-1]:surfaceidx[i]])
  }
```

```
}

if (tail.idx==1) dummy1[dummy.L] <- max(X$values[surfaceidx[length(surfaceidx)]:length(X$values)])

########################################
## Dummy variable for Dive Duration ##
########################################

m <- 1 # m is updated according to which "dive" we are inside

# loop over X lengths, and update accordingly
for(k in 1:length(X$lengths)){
    # if we are at the surface, add the numbers of surface ticks
    if(X$values[k]==0){
     if (k!=1){m <- m+1} # new "surface dive"
     dummy2[m] <- X$lengths[k]
     m <- m+1 # begin new "other" dive
    }
    # else, we are diving and we add the time (length) of the specific depth to
    # the dive duration (TDD)
    else{
      dummy2[m] <- dummy2[m] + X$lengths[k]
    }
  }

##################################################################
## USE DUMMY variables to UPDATE Target Depth and Dive Duration ##
##################################################################
df$TD <- rep(dummy1,dummy2)
df$TDD <- rep(dummy2,dummy2)

# remove unused column
df$depth <- NULL

##################################
## Label Target depth intervals ##
##################################

# quantiles (in meters of 20) used to section Target Depth
qts <- c(1,6,15)
# add "TDsec" variable which signals in which Target Depth area the whale
# appear: Shallow (Sh),Medium (M), Deep (D) and Surface (S)
df <- mutate(df, TDsec = ifelse(TD>15,"D",
                                ifelse(TD>6,"M",
                                       ifelse(TD>1,"Sh","S"))))

# display
head(df)
```

```
##   ind min fineMetric covariate          Q90        Q50 depthlvl TD TDD TDsec
## 1   1   1   4.395885         B 3.905394e+16 8650.39989        3  3  11    Sh
## 2   1   2   4.815291         B 2.004466e+11  669.08492        3  3  11    Sh
## 3   1   3   6.505648         B 4.541147e+08  186.08175        3  3  11    Sh
## 4   1   4   6.092357         B 1.175617e+07   86.34502        2  3  11    Sh
```

```
## 5   1   5   8.469809         B 1.028804e+06   51.75190         2  3  11    Sh
## 6   1   6  12.492105         B 1.805784e+05   35.90317         2  3  11    Sh
```

### Construct Reference Distribution

We now introduce the code, used to generate the (weighted) reference distribution **Q**.

```
tN <- 40 # conservative guess at normal area

##############################################################
## Define weights used to calculate reference distribution ##
##############################################################

# vector of drop (time) points
tdrop <- sort(round(aggregate(df[df$min>tN*60,]$min,
                        list(df[df$min>tN*60,]$ind),
                        function(x)length(x)/60)$x))+tN
nwhales <- length(unique(df$ind)) # number of whales
nt <- nwhales + 1 - cumsum(rle(tdrop)$lengths) # number of whales before each drop point
tdrop <- rle(tdrop)$values # remove dublicates
tdist <- tdrop-shift(tdrop,1) # distance between drops
tdist[1] <-tdrop[1]-tN+1
tdistrel <- tdist/sum(tdist) # relative distance

# weights
Qweights <- nt/sum(nt)

########################################
## Construct reference distribution Q ##
########################################

constructQ <- function(dtIn,R=0){
  # Dataframe and R (boolean) for random sampling where
  # we exclude a random subset of size M if R =1

  ndrops <- length(tdrop) # number of drops
  numberOfTDsections <- length(qts) # number of target depth intervals

  # calculate Q at areas with diffeent weight
  # (Dimension = Target depth sections x Number of drop points)
  Qmat <- matrix(0,nrow=numberOfTDsections,ncol=ndrops)

  tmax <- max(tdrop) # last whale droptime

  # dummy variables
  dummyvec <- rep(0,ndrops)
  tdist.dummy <- tdist

  # select random hourly interval in normal area to exclude
  # for cross validation
  if(R<0)tskip <- sample(tN:(tmax),1)
  # ... or select specific hourly interval to exclude
  if(R>0)tskip <- R

  # adjusted normalization after removal of a single hourly interval
```

```r
  idxRemove <- ndrops+1-sum(tskip<=tdrop)
  tdist.dummy[idxRemove] <- tdist.dummy[idxRemove]-1

  Q <- rep(0,length(qts)-1) # normal distribution vector
  for(t in tN:(tmax)){

    if(t!=tskip){ # if not the random subset that we exclude
      # if the whale has left the surface
      if(nrow(dtIn[dtIn$min>=(t-1)*60 & dtIn$min<t*60 & dtIn$TDsec=="S",])!=0){

      t.N <-aggregate(list(TDD=dtIn[dtIn$min>=(t-1)*60
                            & dtIn$min<t*60 & dtIn$TDsec!="S",]$TDD),
                   list(TDsec=dtIn[dtIn$min>=(t-1)*60
                                  & dtIn$min<t*60
                                   & dtIn$TDsec!="S",]$TDsec),
                   sum)


      # get probability at each TD cell
      TDsecIdx <- match(t.N$TDsec,c("D","M","Sh")) # find matches
      t.N0 <- data.frame(TDsec = c("D","M","Sh"),TDD=0)
      t.N0[TDsecIdx,"TDD"] <-t.N$TDD

      # get relevant area
      idx <- ndrops+1-sum(t<=tdrop)

      # update distribution Q with that area
      Qmat[1:numberOfTDsections,idx] <- Qmat[1:numberOfTDsections,idx]+t.N0$TDD/sum(t.N0$TDD)

      }
      else # the whale never leaves the surface in that interval
        {
        # get relevant area
        idx <- ndrops+1-sum(t<=tdrop)
        # update distribution Q with that area
        Qmat[1:numberOfTDsections,idx] <-  Qmat[1:numberOfTDsections,idx] + rep(0,numberOfTDsections)
        }
     }
  }

  # normalize
  Qmat <- t(replicate(numberOfTDsections,Qweights))*Qmat/t(replicate(numberOfTDsections,tdist.dummy))
  return(rowSums(Qmat))
}

# test
constructQ(df,-1)
```

```
## [1] 0.70089012 0.27384772 0.02526216
```

### Get hourly distributions and hourly Jensen-shannon divergence

We now perform a time loop, and generate the hourly distributions, from which we derive hourly divergence.

```r
##################################################
## Kullback Leibler & Jensen-Shannon function ##
##################################################

Kullback <- function(p,q){
  # Kullback Leibler divergence
  # if p(X)=0,then KB contribution is zero (per definition; see
  # wiki and Kullback Leibler Definition):
  sum(p*log(p/q),na.rm=T)
}

Jensen <- function(p,q){
  # Jensen Shannon divergence
  mm <- 1/2*(p+q)
  return(1/2*(Kullback(p,mm)+Kullback(q,mm)))
}



#####################################
## Depth Diagram Dataframe (DDdf) ##
#####################################

tmax <- floor(max(df$min)/60) # number of hourly intervals
TDsecLabs <- c("Sh","M","D") # number of Target Depth sections = 3 (not including Surface)
numberOfTDsections <- length(qts) # number of target depth intervals
DDdf <- data.frame(Interval=rep(1:tN,each=numberOfTDsections),
                  TDsec=rep(TDsecLabs,tN), #
                  TDD=rep(0,numberOfTDsections*tN))
# add covariate-identifier (A or B)
DDdf <- rbind(DDdf,DDdf)
DDdf$covariate <- rep(c("A","B"),each=tN*numberOfTDsections)


##################################################################
## Calculate hourly distribution and hourly Jensen-Shannon (JS) ##
##################################################################

JS.A <- rep(0,tmax) # JS for category A whales
JS.B <- rep(0,tmax) # JS for category B whales
JS.all <- rep(0,tmax) # JS for all whales

# add reference distribution to Depthgrid
Q <-constructQ(df,-1) # construct Q by removing a K subset randomly
DDdf[DDdf$Interval==tN & DDdf$covariate=="A",]$TDD <- Q
DDdf[DDdf$Interval==tN & DDdf$covariate=="B",]$TDD <- Q

for(t in 1:tmax){

  if(t>=tN){Q<-constructQ(df,1)} # construct specific subset if t>tN

  ### ALL whales:
      t.all <-aggregate(list(TDD=df[df$min>=(t-1)*60
```

```
                              & df$min<t*60 & df$TDsec!="S",]$TDD),
                  list(TDsec=df[df$min>=(t-1)*60
                                      & df$min<t*60
                                      & df$TDsec!="S",]$TDsec),
                  sum)

      # get probability at each TD cell
      TDsecIdx <- match(t.all$TDsec,c("D","M","Sh")) # find matches
      t.NO <- data.frame(TDsec = c("D","M","Sh"),TDD=0)
      t.NO[TDsecIdx,"TDD"] <-t.all$TDD

      # hourly distribution P
      P.all <- t.NO$TDD/sum(t.NO$TDD)

      # calculate hourly Jensen-Shannon
      JS.all[t] <- Jensen(P.all,Q)

  ### Category A whales:

      t.A <-aggregate(list(TDD=df[df$min>=(t-1)*60
                              & df$min<t*60
                              & df$TDsec!="S"
                              & df$covariate=="A",]$TDD),
                  list(TDsec=df[df$min>=(t-1)*60
                                      & df$min<t*60
                                      & df$TDsec!="S"
                              & df$covariate=="A",]$TDsec),
                  sum)

      # get probability at each TD cell
      TDsecIdx <- match(t.A$TDsec,c("D","M","Sh")) # find matches
      t.NO <- data.frame(TDsec = c("D","M","Sh"),TDD=0)
      t.NO[TDsecIdx,"TDD"] <-t.A$TDD

      # hourly distribution P
      P.A <- t.NO$TDD/sum(t.NO$TDD)

      # calculate hourly Jensen-Shannon
      JS.A[t] <- Jensen(P.A,Q)

      if(t<tN) DDdf[DDdf$Interval==t
                    & DDdf$covariate=="A",]$TDD <- P.A

  ### Category B whales:

       t.B <-aggregate(list(TDD=df[df$min>=(t-1)*60
                              & df$min<t*60
                              & df$TDsec!="S"
                              & df$covariate=="B",]$TDD),
                  list(TDsec=df[df$min>=(t-1)*60
                                      & df$min<t*60
                                      & df$TDsec!="S"
                              & df$covariate=="B",]$TDsec),
```

```
                   sum)

      # get probability at each TD cell
      TDsecIdx <- match(t.B$TDsec,c("D","M","Sh")) # find matches
      t.NO <- data.frame(TDsec = c("D","M","Sh"),TDD=0)
      t.NO[TDsecIdx,"TDD"] <-t.B$TDD

      # hourly distribution P
      P.B <- t.NO$TDD/sum(t.NO$TDD)

      # calculate hourly Jensen-Shannon
      JS.B[t] <- Jensen(P.B,Q)

      if(t<tN) DDdf[DDdf$Interval==t
                      & DDdf$covariate=="B",]$TDD <- P.B

}

# Normal area Jensen-Shannon (combine)
trim.max <- 60 # focus only on time below trim.max
JS.N.A <- JS.A[tN:trim.max]
JS.N.B <- JS.B[tN:trim.max]
JS.N <- JS.all[tN:trim.max]

# Weighted JS normal (combining categories)
JS.N <- c(JS.N.A,JS.N.B)
```

### Visualizing depth diagram

From the Depth Diagram data frame (DDdf) we can easily visualize the hourly distributions:

```
intLabsx <- c(1:(tN-1),"Normal") # hourly intervals
# target depth intervals:
intLabsy <- mapply(function(X,Y)paste0("[",X,",",Y,"[") ,qts[-N]*20,shift(qts,-1)[-N]*20)
intLabsy[length(qts)] <- paste0(">",qts[length(qts)]*20)
names(intLabsy)<-NULL
names(qts)<- NULL

# plot
ggplot(DDdf,aes(x=as.factor(Interval),y=as.factor(TDsec),fill=TDD)) +
  geom_tile(aes(fill=TDD),color="black") +
  coord_fixed()+
  xlab("hours")+
  ylab("Target depth (meters)")+
  scale_fill_gradient(low = "white", high = "gray0",name="Time spend (%)")+
  scale_x_discrete(breaks = 1:tN,labels=intLabsx)+
  scale_y_discrete(breaks=c("Sh","M","D"),labels=intLabsy)+
  theme(axis.text.x = element_text(angle = 45, vjust = 1.2, hjust=1),
        plot.title =element_text(hjust = 0.5))+
  ggtitle("Hourly emperical distribution(s) of Dive Duration for N=10")+
  facet_wrap(.~covariate,ncol=1)
```

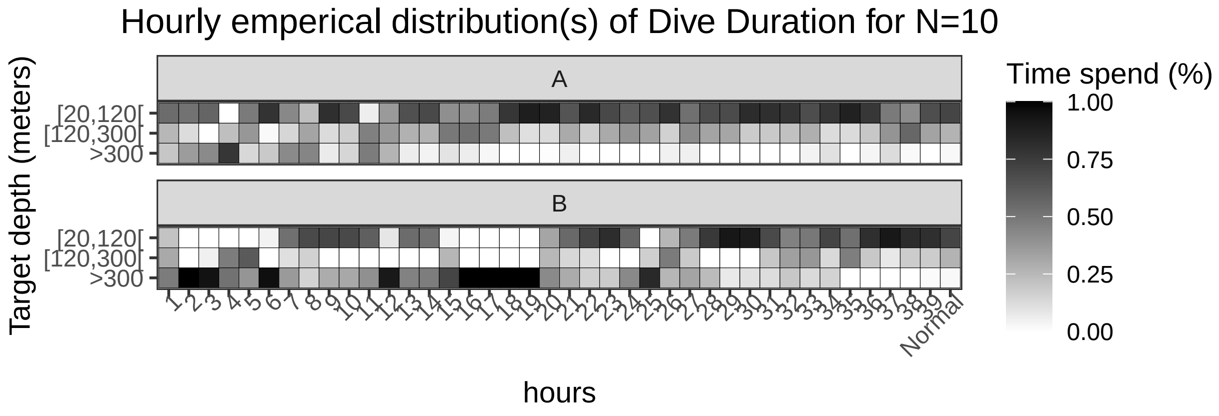


**Block bootstrapping**

We will now walk through the procedure of how block bootstrapping is exploited to produce estimates and confidence bands of recovery times.

Once temporal measures of relative cross entropy has been calculated, denoted $J_t$, we will calculate the 97.5% and 2.5% quantile of $\{J_t \,|\, t > t_N\}$ and denotes thee $\alpha_u$ and $\alpha_l$ respectively. We then introduce a map $m : \mathbb{R} \mapsto \{-1, 1\}$ fulfilling $m(J_t) = 1$ if $J_t > \alpha_u$ or $J_t < \alpha_l$ and $m(J_t) = -1$ otherwise. Define now $y_{t'} = \sum_{t<t'} m(J_t)$, then we perform block bootstrapping on the set $\{y_1, ..., y_{tmax}\}$ with block width equal to roughly $tmax^{1/3}$ (common rule in litterature), and for each sample we estimate a breakpoint using segmented regression (the package Segmented in R). We create 200 samples, and determine the average breakpoint and calculate the 97.5 and 2.5 quantile to obtain confidence bounds.

The code below, implements the procedure.

```
##########################################
## METHOD: BLOCK BOOTSTRAPPING SIMPLE  ##
##########################################

alpha.lower <- quantile(JS.N,0.025) # lower bound
alpha.upper <- quantile(JS.N,0.975) # upper bound

# Function to check if inside or outside CI
plusMinus <- Vectorize(function(x) ifelse(x>alpha.upper | x < alpha.lower,-1,1))

N.sims <- 200 # number of simulations
lB <- 5 # length of block
```

```r
B <- floor(trim.max/lB) # number of blocks
t.max <- floor(trim.max/lB)*lB # maximum hours
time.hrs <- 1:t.max # time (vector) in hours

# breakpoints
BP.A <- rep(0,N.sims) # category A
BP.B <- rep(0,N.sims) # category B

# sample for graphical example
exN <- 3
exdf.A <- data.frame(hours=rep(time.hrs,exN),
                     votes=NA,
                     ID=rep(1:exN,each=t.max))
exdf.B <- data.frame(hours=rep(time.hrs,exN),
                     votes=NA,
                     ID=rep(1:exN,each=t.max))

for(n in 1:N.sims){
  # case resampling within each block
  V.bs.A <- rep(0,t.max)
  V.bs.B <- rep(0,t.max)
  for(b in 0:(B-1)){
    idx <- (b*lB+1):((b+1)*lB) # indices
    V.bs.A[idx] <- sample(plusMinus(JS.A[idx])) # block sampling
    V.bs.B[idx] <- sample(plusMinus(JS.B[idx])) # block sampling
  }
  # make dataframes
  datf.A <-data.frame(votes=c(0,cumsum(V.bs.A)),hours=c(0,time.hrs))
  datf.B <-data.frame(votes=c(0,cumsum(V.bs.B)),hours=c(0,time.hrs))

  # save graphical example
  if(n <= exN){
    exdf.A[exdf.A$ID==n,]$votes <- cumsum(V.bs.A)
    exdf.B[exdf.B$ID==n,]$votes <- cumsum(V.bs.B)
  }

  # compute breakpoints using segmented regression
  lm.A <- lm(votes~hours,data=datf.A)
  sm.A <- segmented(lm.A)
  lm.B <- lm(votes~hours,data=datf.B)
  sm.B <-segmented(lm.B)

  BP.A[n] <- sm.A$psi[2]
  BP.B[n] <- sm.B$psi[2]
}

# estimates and CI
est.A <- mean(BP.A) # recovery A
CI.A <- quantile(BP.A,c(0.025,0.975)) # CI A
est.B <- mean(BP.B) # recovery B
CI.B <- quantile(BP.B,c(0.025,0.975)) # CI B
```

```r
######################################
## Visualize example of bootstrap ##
######################################
exdf <- rbind(exdf.A,exdf.B)
exdf$category <- rep(c("A","B"),each=t.max*exN)

# plot graphical examples
ggplot(exdf, aes(hours,votes,color=factor(ID)))+
  geom_line(size=1)+
  facet_wrap(.~category)+
  ggtitle("3 block bootstrap samples for each category (A and B)")
```

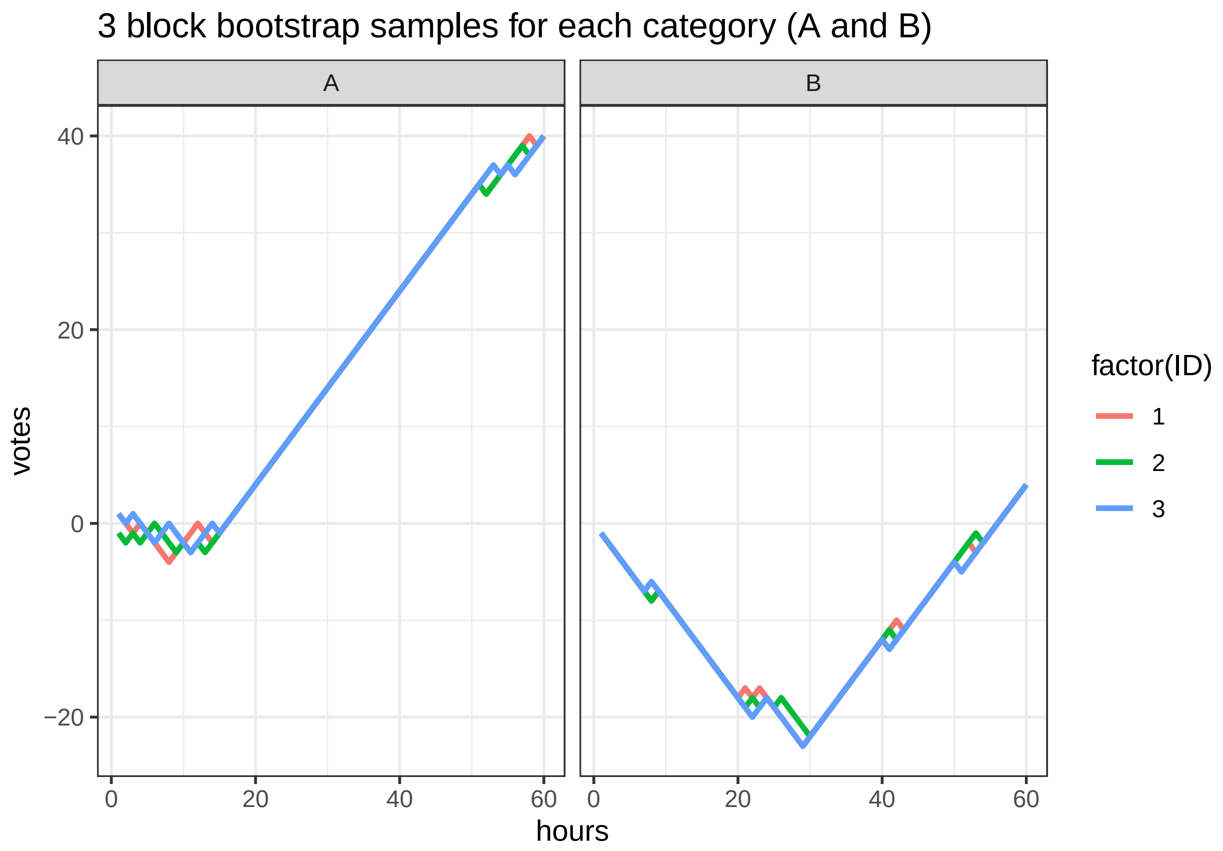


The plots above show 3 bootstrap samples. They all drop initially, suggesting that the first temporal blocks only produce negative values in the bootstrapping which means that there are no RCE measurements inside the RoR. They rise sporadically, because few measurements falls inside the RoR, and eventually rise steadily as measurements begin to stabilize inside the RoR. The (small) variation in the samples means that segmented regression will either push back or push forward the breakpoint estimate to obtain the best fitting model.

### Jensen-Shannon divergence

Finally we are ready to produce the Jensen-Shannon divergence figures, using the Jensen-Shannon measures and the bootstrap estimates.

```r
# Differentiate handling time, and weight JS-normal
df.JS <- data.frame(Interval=rep(1:trim.max,2),
                    category=rep(c("A","B"),each=trim.max),
JS=c(JS.A[1:trim.max],JS.B[1:trim.max]))
```

```r
plot.max <- trim.max # plotting range

ggplot(df.JS,aes(x=Interval,y=JS,fill=category))+
geom_ribbon(aes(ymin=quantile(JS.N,0.025) ,alpha=I(0.2),
                ymax =quantile(JS.N,0.975)),
            color="black",fill="thistle")+
  geom_point(size=2,shape=24)+
  geom_vline(aes(xintercept=est.B),linetype="longdash",color="red")+
  geom_vline(aes(xintercept=CI.B[1]),linetype="solid",color="red")+
  geom_vline(aes(xintercept=CI.B[2]),linetype="solid",color="red")+
  geom_vline(aes(xintercept=est.A),linetype="longdash",color="blue")+
  geom_vline(aes(xintercept=CI.A[1]),linetype="solid",color="blue")+
  geom_vline(aes(xintercept=CI.A[2]),linetype="solid",color="blue")+
  geom_hline(aes(yintercept=mean(JS.N)),linetype="longdash")+
  ylab("Jensen-Shannon divergence")+
  xlab("hours")+
  scale_x_continuous(breaks=seq(0,plot.max,5),
                     labels=seq(0,plot.max,5),
                     limits=c(1,plot.max))+
 scale_color_manual(values=c("blue", "red"))+
  scale_fill_manual(values=c("royalblue", "brown1"))+
  labs(title = "Cross entropy of hourly Dive duration
       post-tagging relative to normal (> 40 hours)
       Dive duration for N=10 individuals ")+
  theme(legend.position="bottom",plot.title=element_text(hjust=0.5))
```

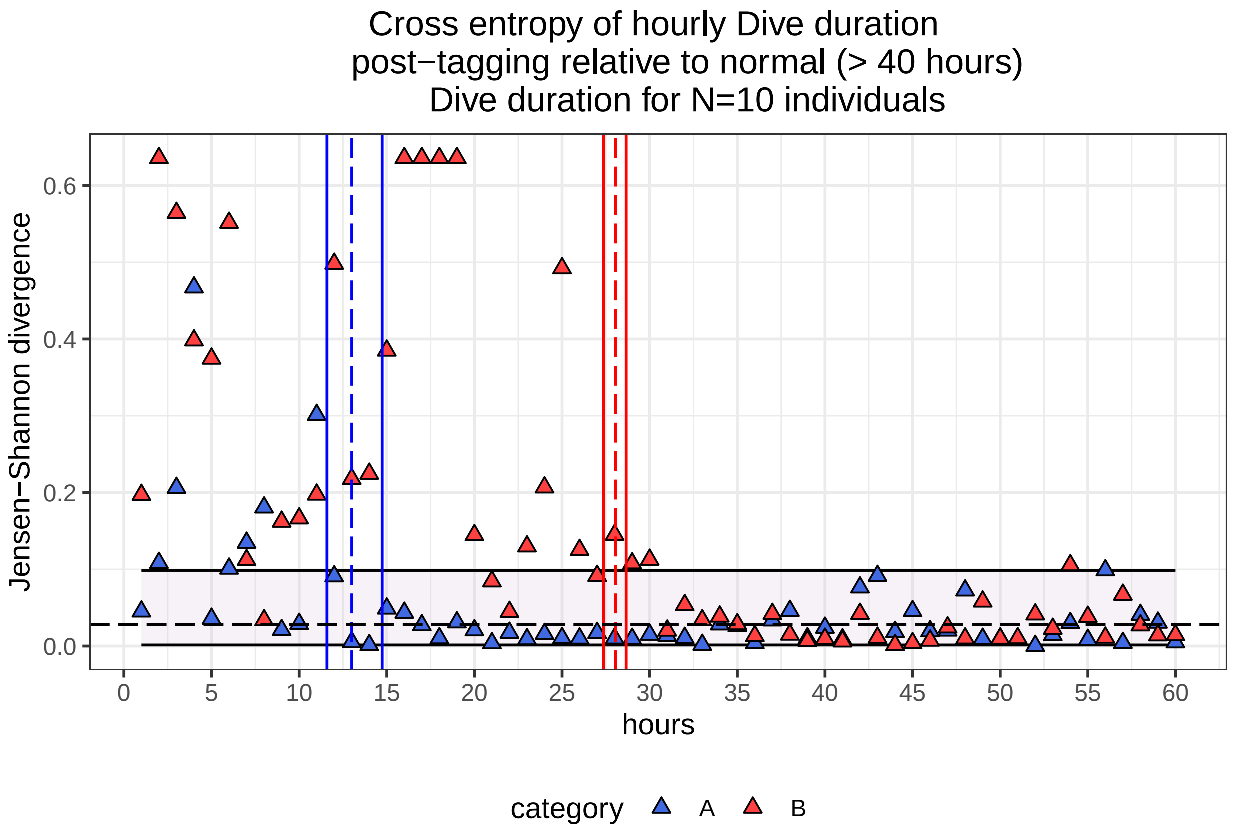

## Supplementary figures (on actual datasets)

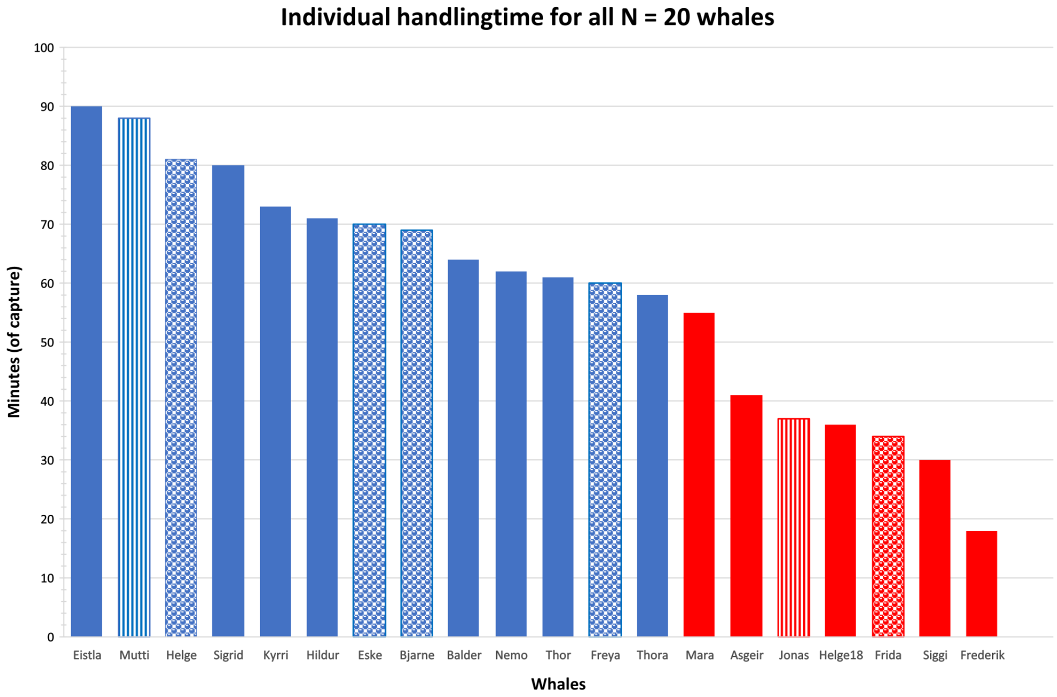


Figure 1: Visual image of relative handling times

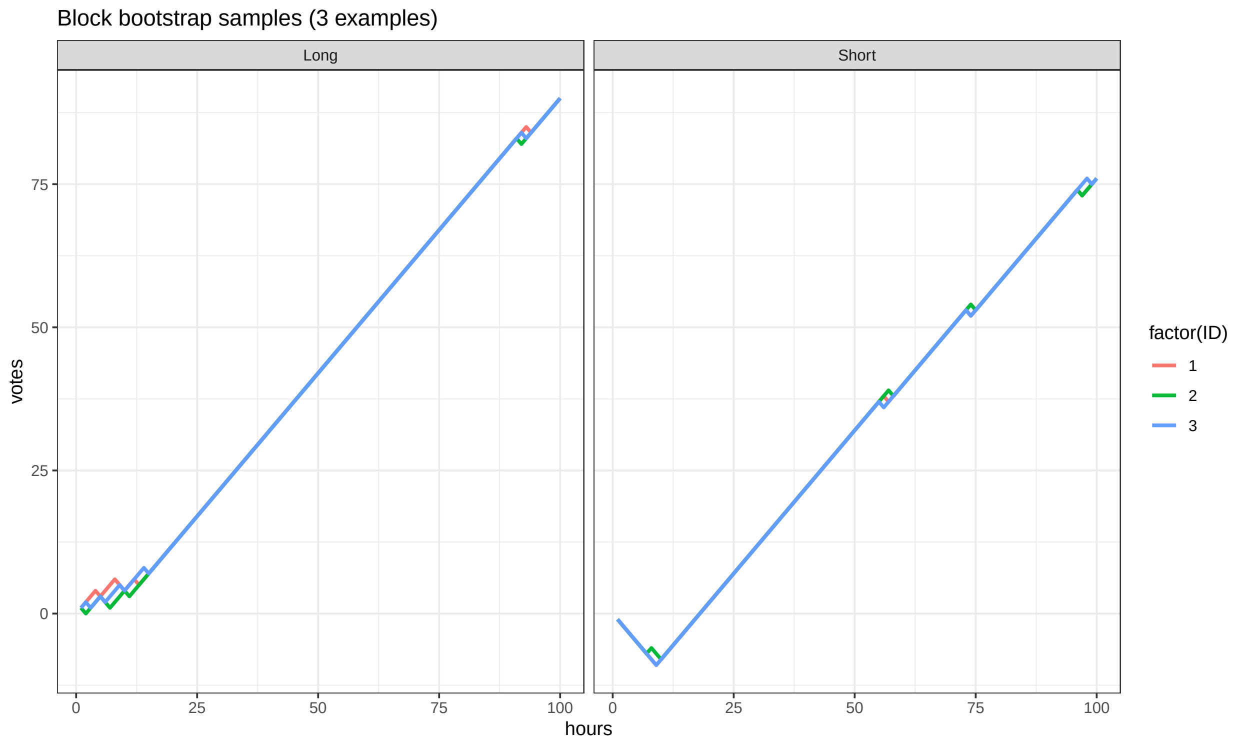


Figure 2: 3 bootstrap examples out of 200 simulations for narwhals

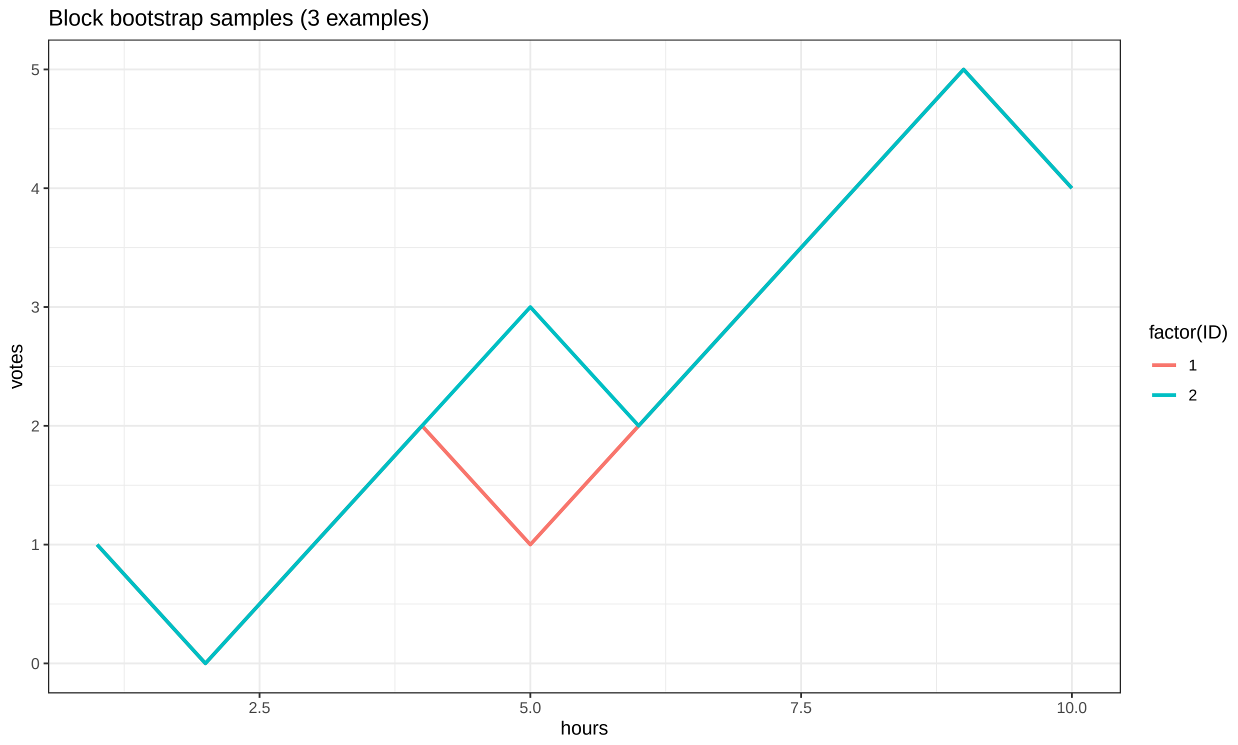


Figure 3: 2 bootstrap examples out of 200 simulations for bowhead whales

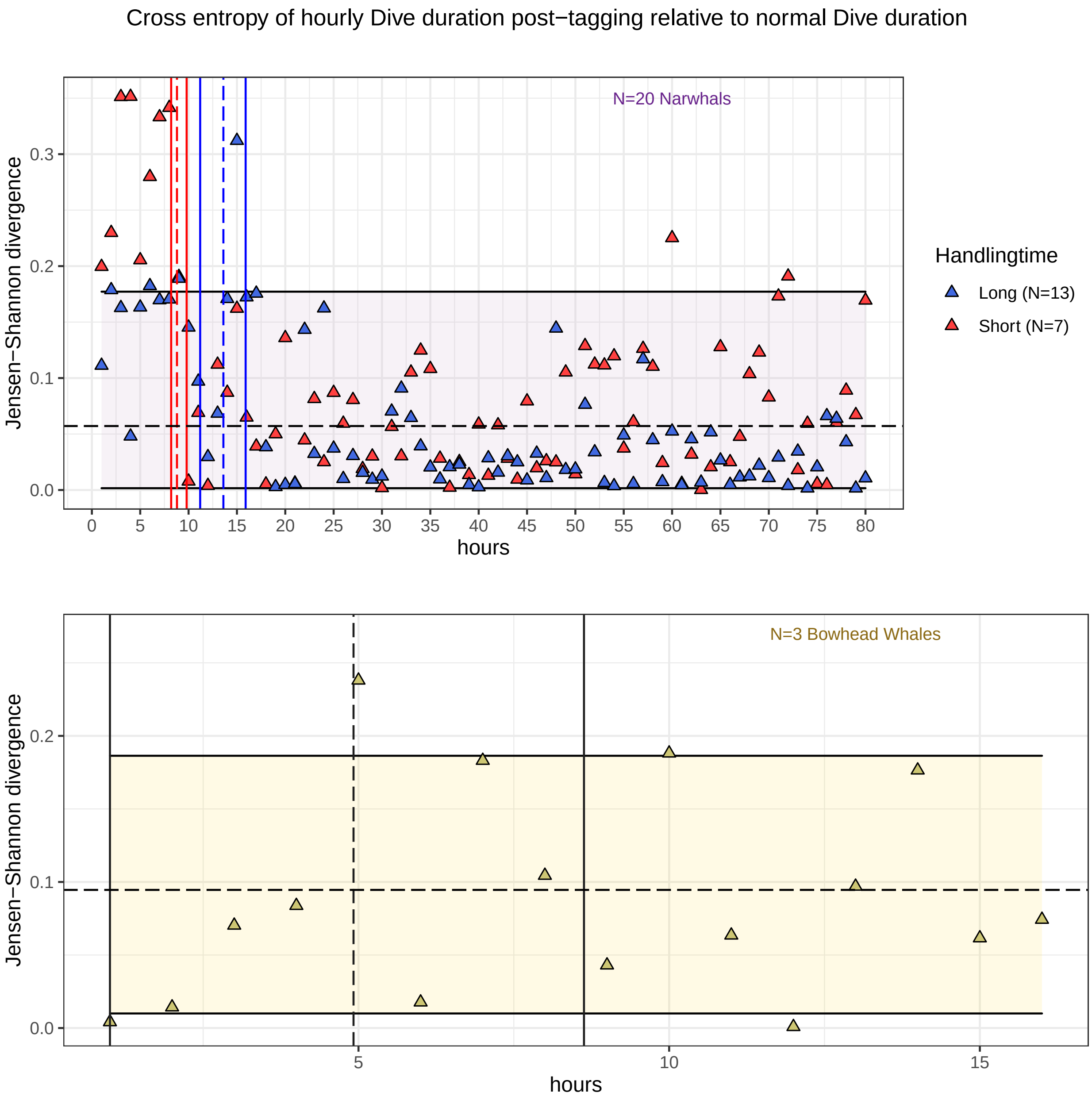


Figure 4: Jensen-Shannon plot from article with local regression smooth

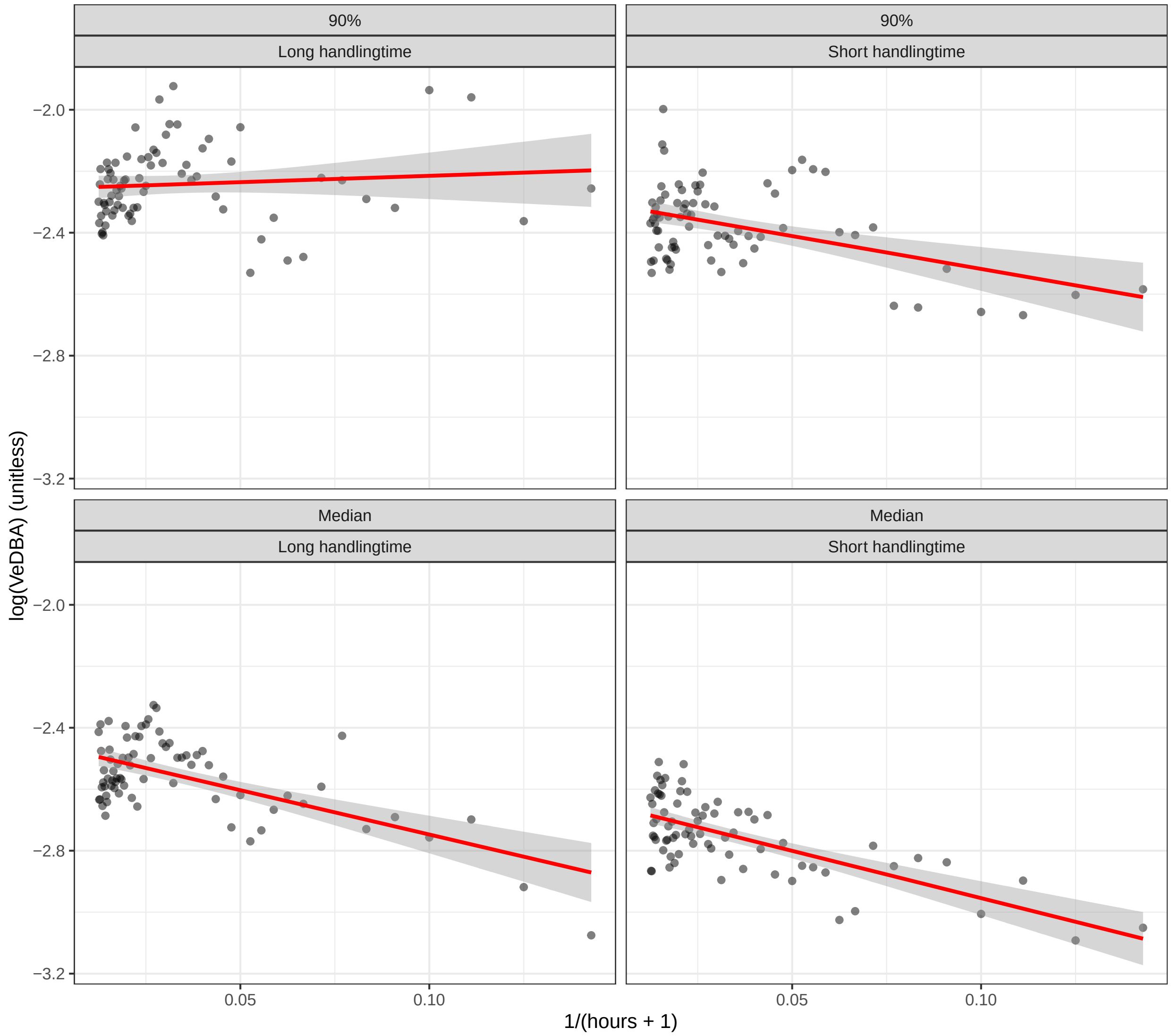


Figure 5: Linearity assumption in QR for narwhals. Focused on segment >5 hours and <50 hours.

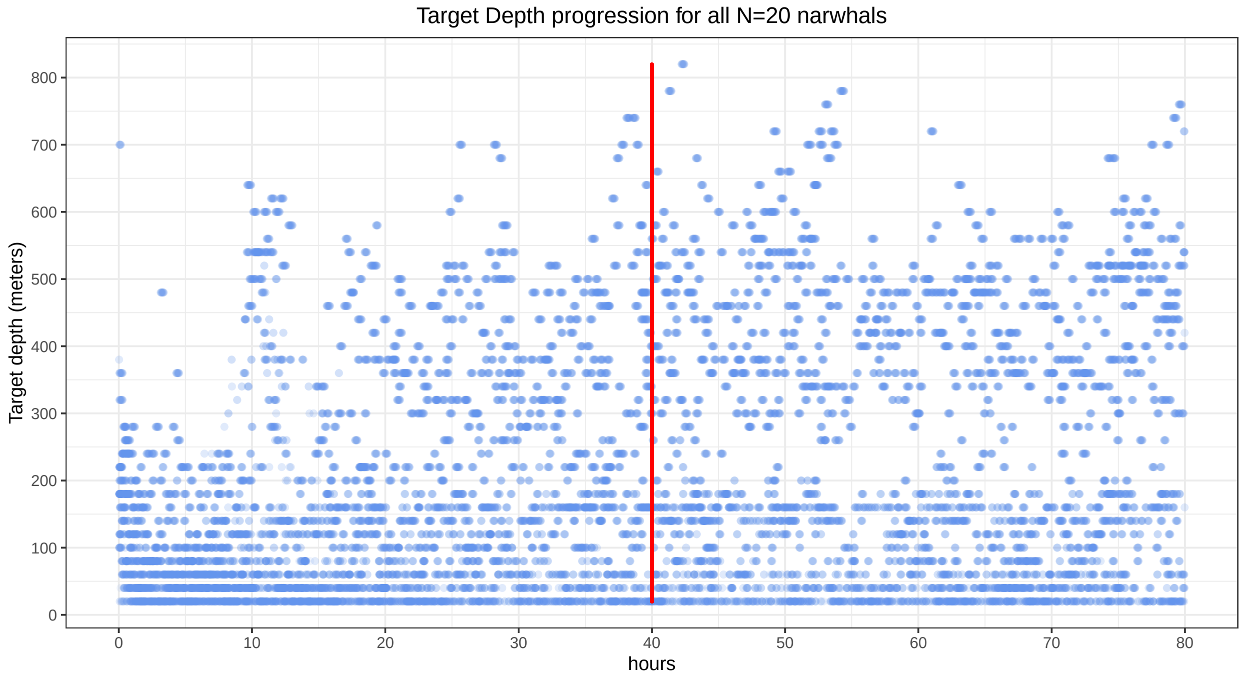


Figure 6: Evolution of target depth for narwhals. Red line t=40 signals the conservative normal area.

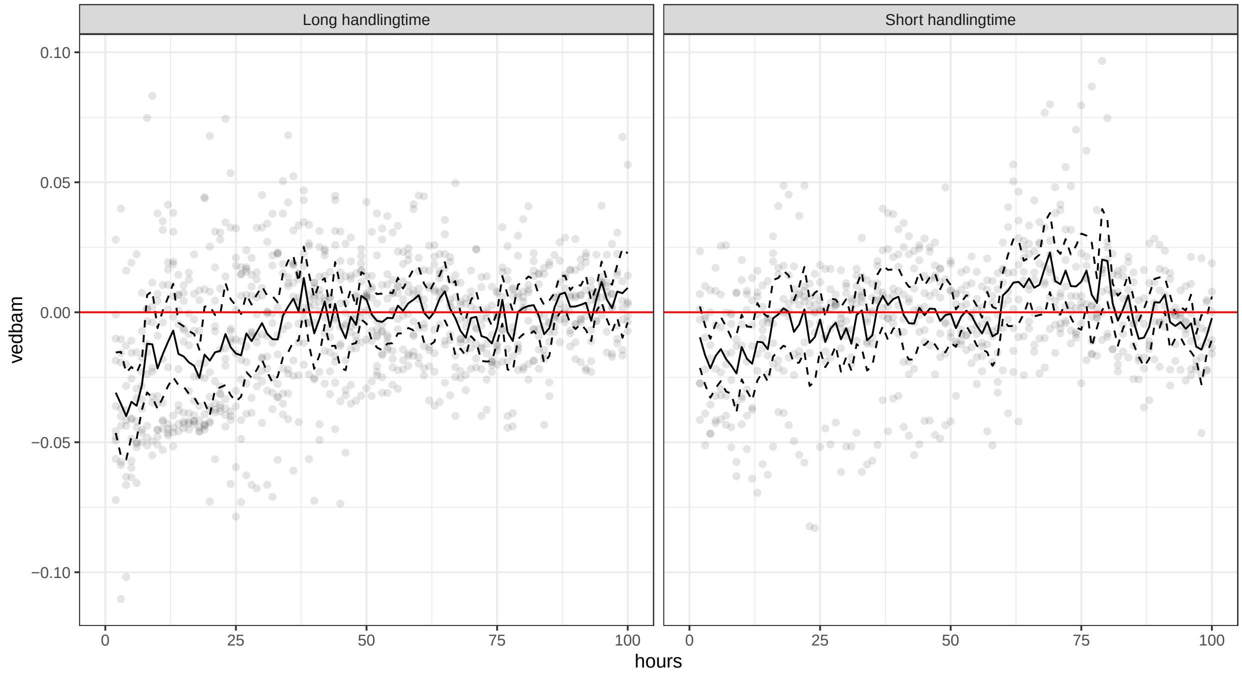


Figure 7: Result of mean-based regression with metric=VeDBA for narwhals

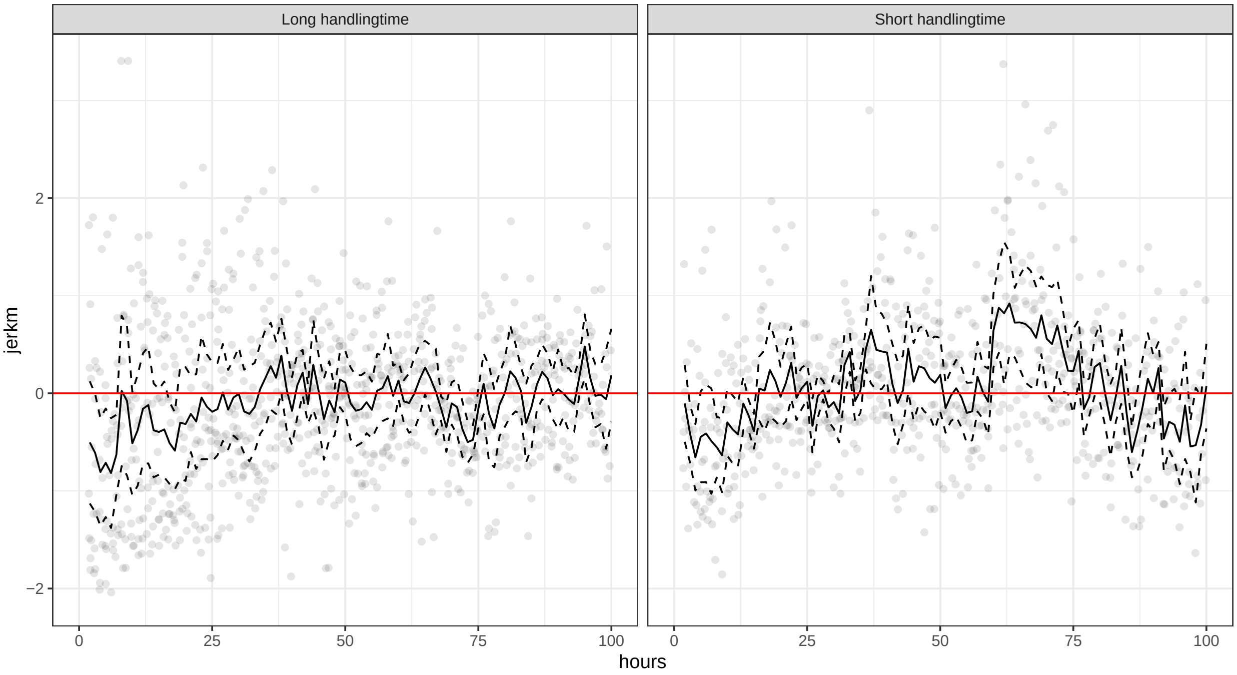


Figure 8: Result of mean-based regression with metric=Jerk for narwhals

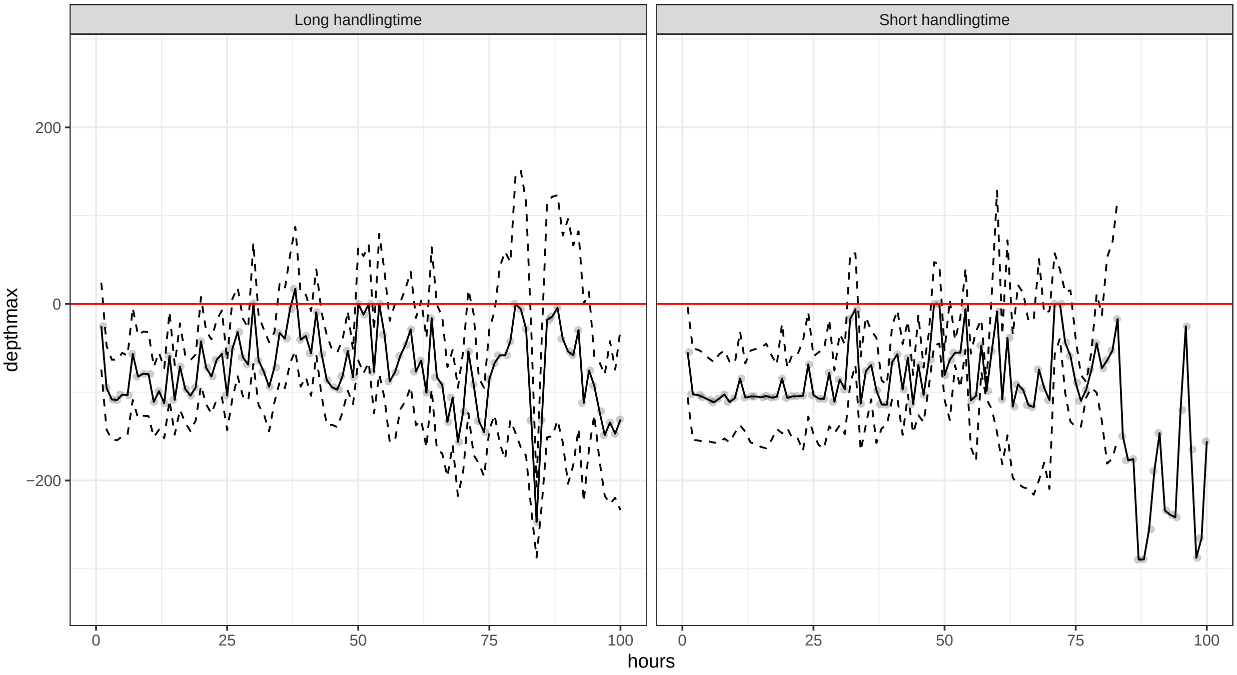


Figure 9: Result of mean-based regression with metric=Max Depth for narwhals

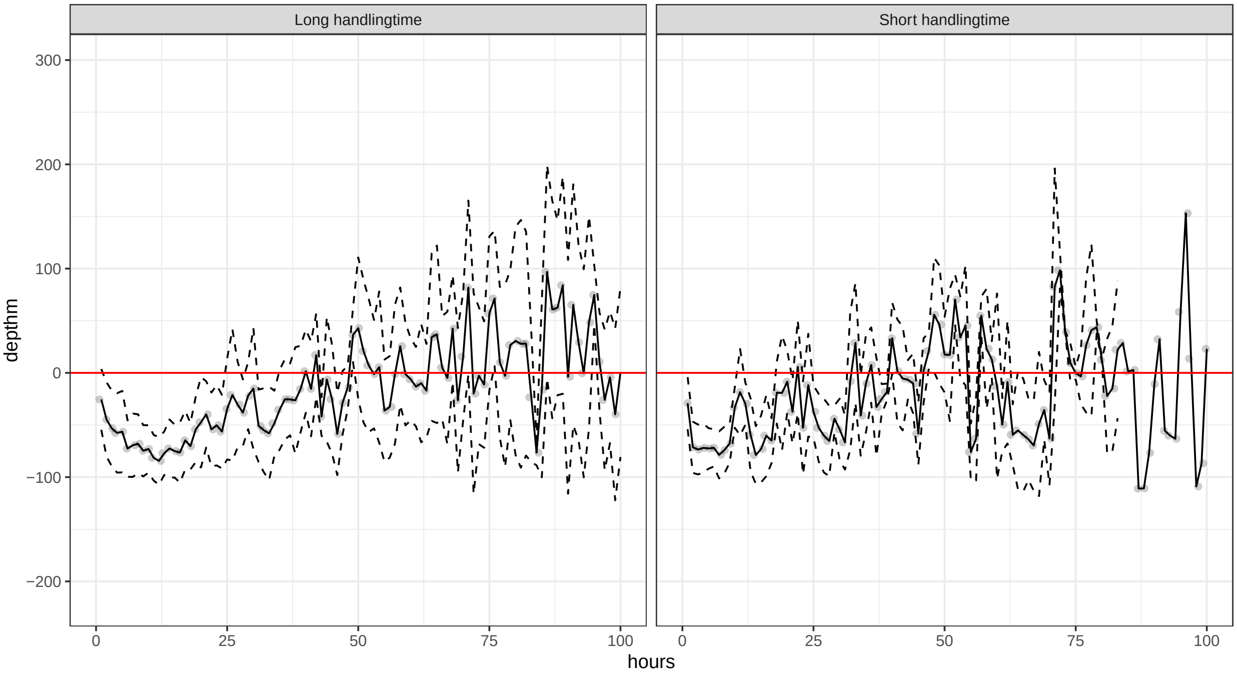


Figure 10: Result of mean-based regression with metric=Mean Depth for narwhals

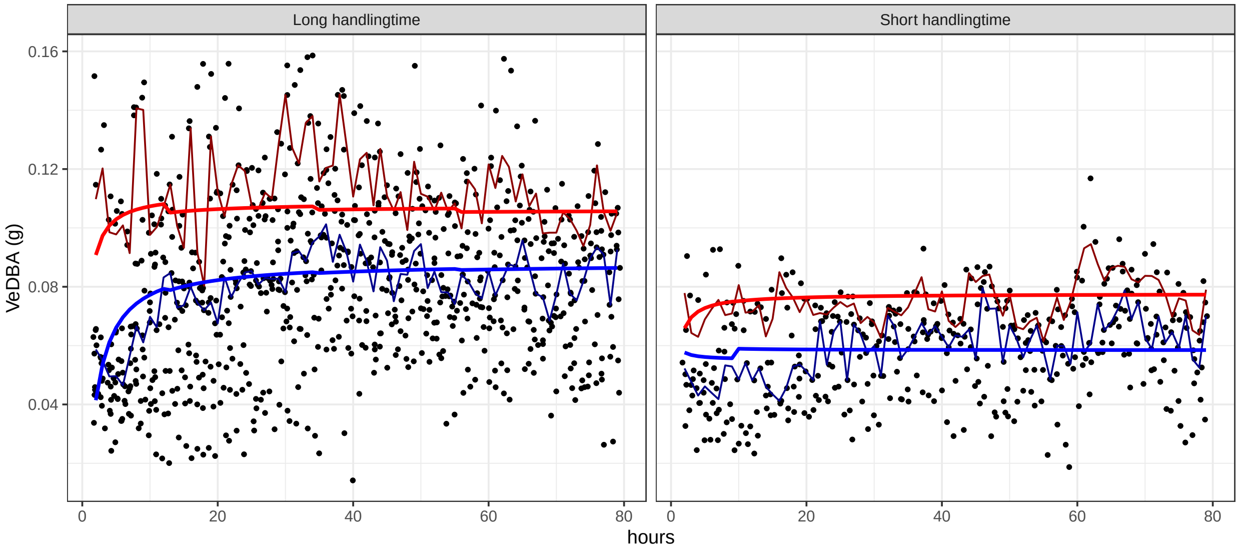


Figure 11: Avg. median and Avg. 90% quantile for VeDBA - Narwhals

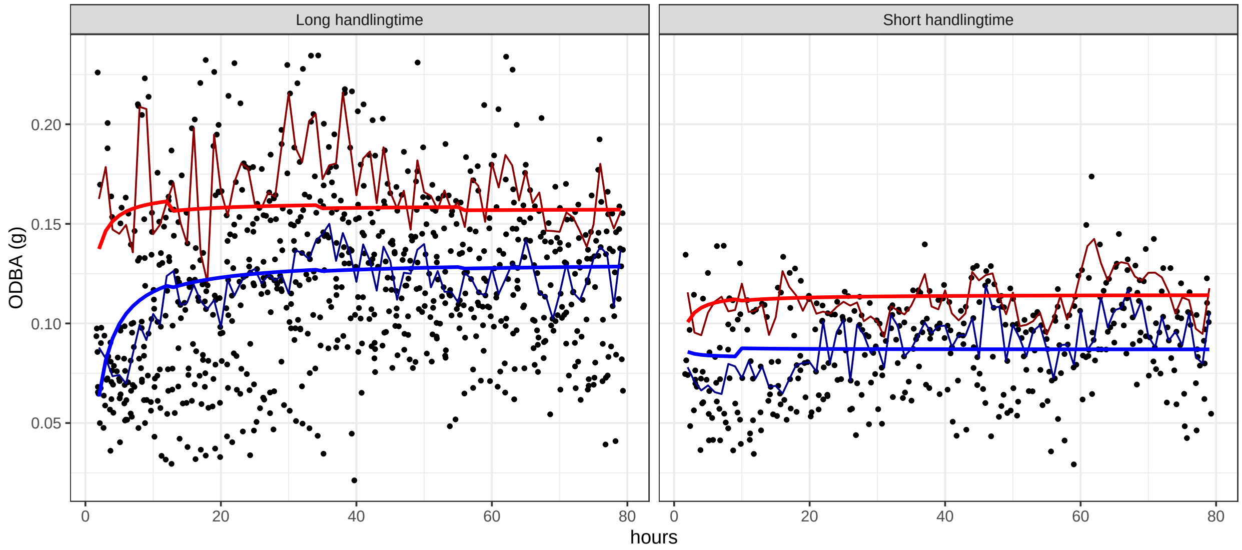


Figure 12: Avg. median and Avg. 90% quantile for ODBA - Narwhals
